# BAT-derived miR-378a-3p facilitates endothelial angiogenic function and promotes wound healing

**DOI:** 10.1172/jci.insight.201311

**Published:** 2026-04-21

**Authors:** Hongyan Deng, Yuyu Xie, Jiadai Liu, Jing Ge, Qianqian Kang, Rui He, Zhihan Wang, Xuemin Peng, Zengzhe Zhu, Wenshe Wang, Yulian Liu, Ronghui Gao, Ruping Pan, Min Yang, Yong Chen

**Affiliations:** 1 Division of Endocrinology, Department of Internal Medicine, Tongji Hospital, Tongji Medical College and State Key Laboratory for Diagnosis and Treatment of Severe Zoonotic Infectious Diseases, Huazhong University of Science and Technology, Wuhan, China.; 2 Laboratory of Endocrinology and Metabolism, Ministry of Education Key Laboratory of Vascular Aging, Tongji Hospital, Tongji Medical College, Huazhong University of Science and Technology, Wuhan, China.; 3 Department of Nuclear Medicine, Tongji Hospital, Tongji Medical College, Huazhong University of Science and Technology, Wuhan, China.; 4 Department of Endocrinology, Fifth People’s Hospital of Shanghai, Fudan University, Shanghai, China.; 5 Branch of National Clinical Research Center for Metabolic Diseases, Wuhan, China.; 6 The Center for Biomedical Research, Tongji Hospital, Tongji Medical College, Huazhong University of Science and Technology, Wuhan, China.

**Keywords:** Metabolism, Vascular biology, Adipose tissue, Angiogenesis, Diabetes

## Abstract

Interscapular brown adipose tissue (BAT), one of the most vascularized tissues in the body, exemplifies the intricate crosstalk between the vascular system and adipocytes. BAT is known to secrete abundant exosomes into circulation, and exosomes are known to play a key role in vascular remodeling and cell migration. However, whether BAT-derived exosomes (BATexos) modulate peripheral vasculature remains unclear. Here, we report that BATexos promoted peripheral angiogenesis and vascular repair. Among their cargo, miR-378a-3p was highly enriched and identified as a key mediator of endothelial angiogenic function. The overexpression of miR-378a-3p in endothelial cells substantially promoted cell migration and tube formation. Conversely, inhibition of exosome secretion from BAT impaired vascular repair and delayed wound healing. Mechanistically, miR-378a-3p directly targeted the phosphatase and tensin homolog (*Pten*), thereby activating the PI3K/AKT signaling pathway. Liposomes encapsulating miR-378 mimics promoted angiogenesis and accelerated wound healing in a diabetic mouse model. Collectively, this study uncovers BAT-derived miR-378a-3p as a key regulator of vessel regeneration and tissue repair after injury, offering therapeutic potential for treating vascular complications in metabolic disease.

## Abstract 2

<div>Brown fat releases a natural signal that helps wounds heal faster by boosting blood vessel formation.</div>

## Graphical abstract

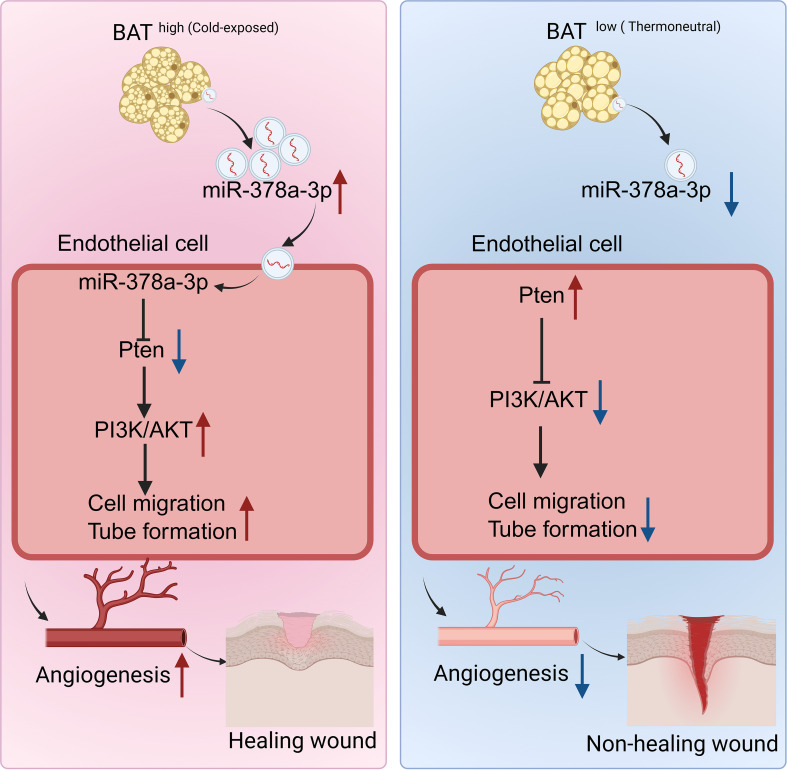

## Introduction

Endothelial cells (ECs), which constitute the innermost lining of blood vessels, play a key role in maintaining vascular system homeostasis and facilitating repair processes (1). The proliferation and migration of ECs are essential steps in neovascularization (2). When vessels are damaged, smooth muscle cells migrate to stabilize the lesion and limit neointima formation, and ECs undergo proliferation and migration — a process known as reendothelialization — to restore vascular integrity. However, during the later stages of wound healing, excessive EC proliferation and migration is known to inhibit smooth muscle cell migration, potentially impairing vessel remodeling. Metabolic and cardiovascular diseases such as hypertension, hyperlipidemia, and diabetes often lead to endothelial dysfunction, contributing to pathological vascular changes such as macrovascular disease (e.g., atherosclerosis) and microvascular complications (e.g., delayed wound healing in diabetes) (3). Improving the angiogenic capacity of ECs is therefore considered a promising strategy to improve vascular regeneration and counteract tissue damage induced by metabolic stress.

Brown adipose tissue (BAT) is a thermogenic tissue that plays a pivotal role in maintaining core body temperature (4). In addition to its thermogenic functions, BAT is also a key endocrine organ, secreting a wide array of cytokines (BATokines) and exosomes (BATexos) in response to physiological stressors to regulate systemic metabolic homeostasis (5–7). BAT is richly vascularized, containing dense capillary networks that support its high metabolic and thermogenic activity (8). Cold exposure stimulates vascular density and blood flow in BAT to support thermogenesis and maintain thermal homeostasis (8–10). Recent studies have shown that individuals with vascular disease exhibit reduced BAT activity (11). BATokines may contribute to promoting metabolic health and reducing the risk of vascular disease. For example, human antigen R (HuR), a BAT-derived cytokine, has been shown to improve endothelial function, lower blood pressure, and alleviate vascular remodeling in murine models (6). Furthermore, BATexos have been implicated in mitigating aging-related fertility decline and cardiometabolic disorders (12, 13). These findings suggest a strong correlation between BAT activity and vascular health; however, whether BATexos directly modulate peripheral vascular remodeling remains unclear.

In this study, we revealed that miR-378a-3p, which was highly enriched in BATexos, mediated the effects of BATexos on endothelial angiogenic function and vascular healing. Loss of miR-378a-3p impaired both vascular repair and angiogenesis induced by ECs. Mechanistically, miR-378a-3p promoted endothelial angiogenic function by directly targeting *Pten* and activating the PI3K/AKT signaling pathway. These data identify miR-378a-3p as a critical mediator of the pro-angiogenic effects of BATexos, thereby establishing a key regulatory axis in peripheral vascular regeneration.

## Results

### Ablation of BAT impairs vascular repair and wound healing.

**Figure 1:**
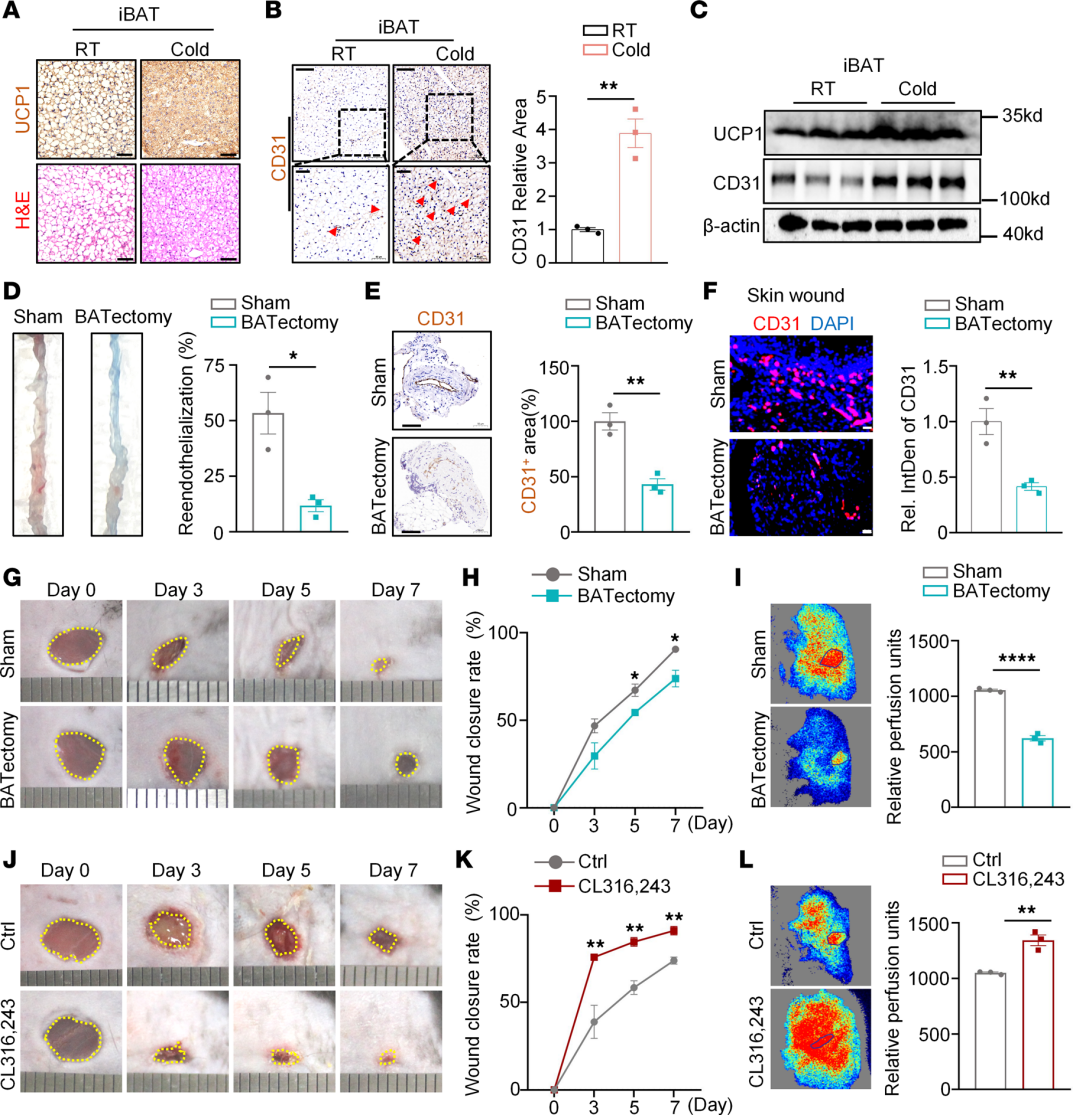
Ablation of BAT impairs vascular repair and wound healing. (**A**) H&E and UCP1 IHC staining of BAT from cold-exposed WT mice. Scale bar: 50 μm. (**B**) CD31 IHC staining in BAT after cold exposure with quantification of CD31+ area. Scale bar: 100 μm (top), 50 μm (bottom). (**C**) Western blot of UCP1 and CD31 in BAT after cold exposure. (**D**) Representative images of Evans blue staining of injured femoral artery in the sham and BATectomy mice (*n* = 3). Quantification data of percentage of reendothelialization are shown on the right. (**E**) Representative images of femoral arteries with CD31 IHC staining in the sham and BATectomy mice (*n* = 3). Scale bar: 50 μm. Right: quantification of the data. (**F**) Immunofluorescence staining of CD31 (red) in wounds of BATectomy and sham mice (*n* = 3). Scale bar: 50 μm. Right: quantification of the data. (**G**) Picture of back skin wounds in BATectomy and sham mice. (**H**) Quantification data of wound closure rate over time in BATectomy and sham mice (*n* = 3–4). (**I**) Representative images of blood flow at the wounds in BATectomy and sham mice. Calculated wound microvascular perfusion is shown on the right (*n* = 3). (**J**) Representative pictures of skin wounds in control and CL316,243-injected mice (*n* = 3–4). (**K**) Quantification data of wound closure rate over time in control and CL316,243-injected mice (*n* = 3–4). (**L**) Representative images of blood flow at the wounds in control and CL316,243-injected mice. Calculated wound microvascular perfusion is shown on the right (*n* = 3). The value n represents the number of biologically independent samples, from which all experimental data were obtained. The data are expressed as mean ± SEM (analyzed by 2-tailed Student’s t test). *P < 0.05, **P < 0.01, ****P < 0.0001.

To investigate the association between BAT activity and angiogenesis, WT mice were exposed to 4°C for 3 days to activate BAT. Cold exposure significantly increased UCP1 expression in BAT (Figure 1A, Figure 1C, and Supplemental Figure 1A; supplemental material available online with this article; https://doi.org/10.1172/jci.insight.201311DS1), along with a marked upregulation of CD31 (encoded by *Pecam1)*, an EC marker (Figure 1, B and C, and Supplemental Figure 1A), indicating a striking correlation between BAT activation and increased local vascularization. To investigate the role of BAT in angiogenesis, we analyzed cold-induced gene expression changes in mouse BAT using dataset GSE280981. By focusing on upregulated genes, we found substantial enrichment of pathways related to vesicle-mediated transport and angiogenesis (Supplemental Figure 1B). Next, to directly assess the role of BAT in vascular repair, we established 2 models: surgical removal of interscapular BAT (BATectomy) and pharmacological activation of BAT using the β3-adrenergic receptor agonist CL316,243 (Supplemental Figure 1C). Endothelial angiogenic function was assessed by monitoring subcutaneous angiogenesis in a dorsal skin wound model and reendothelialization after femoral artery injury, as previously described (14, 15). Compared with sham-operated controls, BATectomy mice exhibited a significantly reduced reendothelialization rate in injured femoral arteries, as demonstrated by Evans blue staining (Supplemental Figure 1D and Figure 1D). CD31 IHC also revealed fewer ECs in the femoral arteries of BATectomy mice (Figure 1E). In addition, CD31^+^ immunofluorescence in wound tissue was reduced in BATectomized mice relative to controls, indicating impaired angiogenesis (Figure 1F). Notably, BATectomy mice showed delayed wound healing compared with sham mice at day 5 (Figure 1, G and H). Consistently, BATectomy significantly reduced microvascular perfusion at the wound site (Figure 1I). Moreover, activation of BAT by β3-adrenergic receptor agonist CL316,243, as evidenced by the marked upregulation of *Ucp1* mRNA and UCP1 protein levels (Supplemental Figure 1, E and F), significantly accelerated wound closure by day 3 compared with vehicle-treated mice (Figure 1, J and K). Also, this coincided with enhanced microvascular perfusion after CL316,243 injection (Figure 1L and Supplemental Figure 1, G and H). Interestingly, serum CD63 concentration was significantly reduced after BAT ablation, as revealed by ELISA (Supplemental Figure 1I). Collectively, these results demonstrate that BAT positively regulates angiogenesis and promotes wound healing, likely via modulation of local vascular density and perfusion.

### BATexos facilitate vascular repair and wound healing.

**Figure 2:**
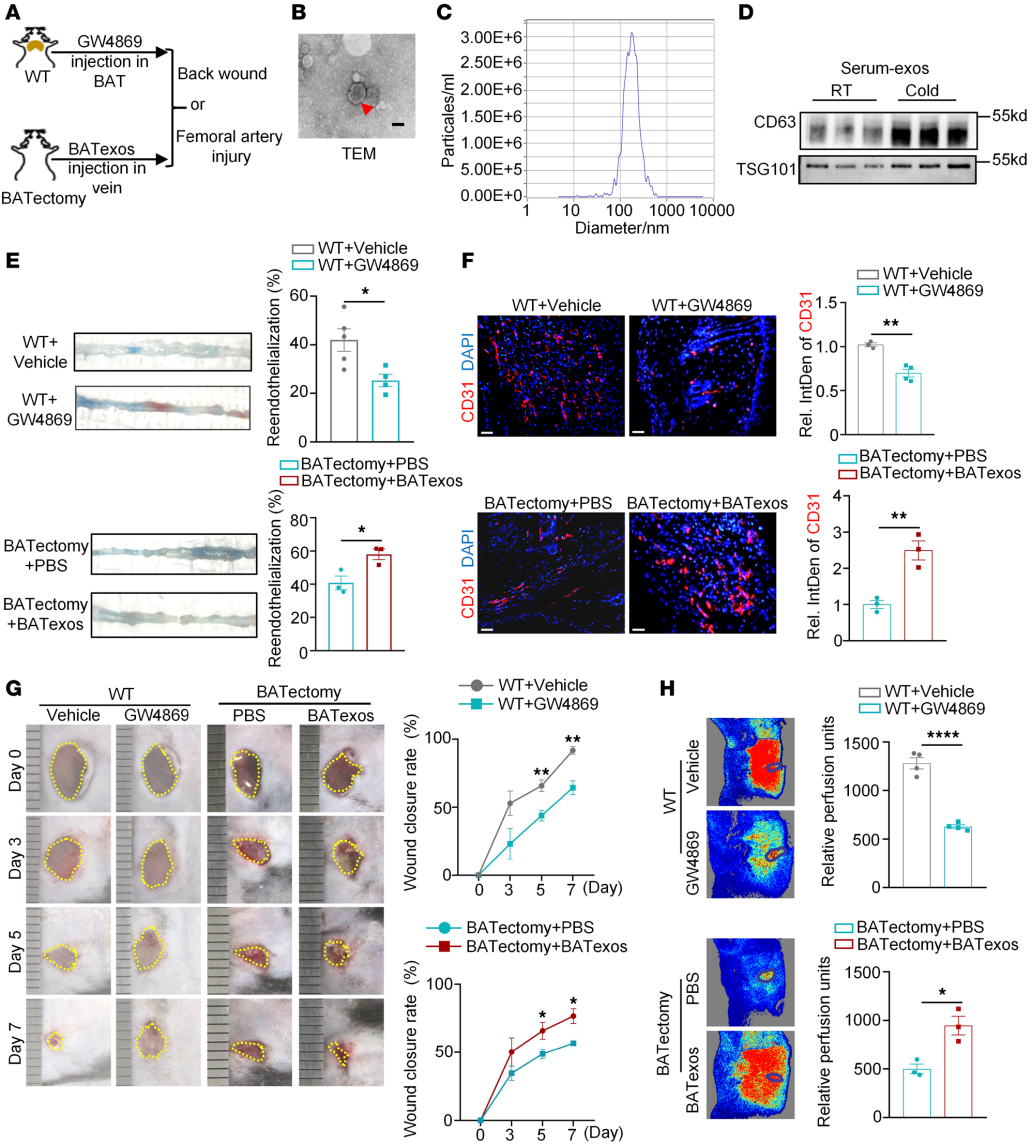
BATexos facilitate vascular repair and wound healing. (**A**) Schematic diagram of BAT with GW4869/control vehicle injection in WT mice or BATexos/PBS treatment in BATectomy mice. (**B**) Morphological characteristics of BATexos under a transmission electron microscope. Scale bar: 100 nm. (**C**) The diameter of the BATexo particles was investigated via nanoparticle tracking analysis. (**D**) Western blot analysis of specific marker proteins (CD63 and TSG101) for exosomes in the serum of mice kept at room temperature or cold (at 4°C). (**E**) Evans blue staining and quantitative analysis of the femoral artery in WT mice injected with GW4869 in BAT or BATectomy mice injected with BATexos via the tail vein. (**F**) Immunofluorescence staining for CD31 (red) in the wound and quantitative analysis in mice treated with GW4869 or BATexos (*n* = 3–4). Scale bar: 50 μm. (**G**) Representative images of skin wounds in the GW4869/vehicle-treated WT mice and BATexos/PBS-treated BATectomy mice. Quantification of wound closure rate presented as a line graph on the right. (**H**) Representative images of blood flow at the wounds in vehicle/GW4869-treated WT mice and PBS/BATexos-treated BATectomy mice. The calculated wound microvascular perfusion is shown on the right (*n* = 3–4). The value *n* represents the number of biologically independent samples, from which all experimental data were obtained. Statistical significance was assessed by 2-tailed Student’s *t* test. The data are expressed as mean ± SEM. *P < 0.05, **P < 0.01, ****P < 0.0001.

Activated BAT is known to secrete exosomes into the circulation (5, 16). To investigate whether BATexos contribute to wound healing and vascular repair, we locally injected the exosome secretion inhibitor GW4869 (100 μL) into the BAT of WT mice to block exosome release (Figure 2A). In addition, we isolated exosomes from cultured brown adipocytes of WT mice in vitro and administered them to the BATectomized mice either via tail vein injection or direct injection into the wound area (Figure 2A). To demonstrate that circulating BATexos can reach the wound site and exert their effects, we i.v. injected PKH26-labeled BATexos via the tail vein and detected the presence of PKH26-labeled exosomes at the wound site. PKH26-labeled BATexos were detected in the wound tissue, indicating that these exosomes reached the wound site via the circulatory system to exert their biological functions (Supplemental Figure 2A). The extracted exosomes showed an atypical cup-shaped morphology under a transmission electron microscope (Figure 2B). Nanoparticle tracking analysis showed particle diameters ranging from 100 to 170 nm (Figure 2C). These particles expressed exosomal protein markers such as CD63 and tumor susceptibility gene 101 (TSG101), whose expression was markedly upregulated in serum exosomes extracted from cold-exposed mice (Figure 2D). Functionally, GW4869-mediated inhibition of exosome secretion in BAT significantly reduced reendothelialization of injured femoral arteries, as evidenced by increased Evans blue staining (Figure 2E). Conversely, the impaired reendothelialization observed in BATectomized mice was rescued by systemic administration of BATexos from WT mice (Figure 2E). Similarly, BAT GW4869 treatment reduced CD31 expression in wound tissues, indicating compromised angiogenesis (Figure 2F). This effect was reversed by local administration of BATexos in BATectomized mice, which restored CD31^+^ EC levels in the skin wound (Figure 2F), indicating improved angiogenesis. Consistent with these findings, GW4869 injection in BAT significantly impaired wound healing (Figure 2G), whereas topical BATexos delivery, but not the PBS control, effectively restored wound healing in BATectomized mice (Figure 2G). We next investigated the role of BATexos in wound perfusion. Local injection of GW4869 into BAT significantly reduced blood perfusion at the wound site (Figure 2H). Importantly, BATexos restored wound perfusion in BATectomized mice (Figure 2H). It has been reported that BATexos play a role in systemic metabolic regulation and energy homeostasis (12, 17). To determine whether the pro-angiogenic effect of BATexos is secondary to altered systemic metabolism, we performed a series of metabolic assessments in BATectomized mice receiving BATexos or vehicle during the active wound healing phase (days 3–5). Indirect calorimetry revealed no significant differences between the BATectomized mice receiving BATexos or the vehicle in terms of either oxygen consumption (VO_2_) (Supplemental Figure 2, B and C) or the respiratory exchange ratio (Supplemental Figure 2, D and E). Furthermore, there was no significant difference in food intake between the 2 groups (Supplemental Figure 2F). Consistent with these findings, an i.p. glucose tolerance test showed no improvement in glucose homeostasis upon BATexos administration (Supplemental Figure 2, G and H). Collectively, these data demonstrate that the observed enhancement in wound angiogenesis and perfusion mediated by BATexos occurs independently of changes in whole-body energy metabolism or glucose tolerance. It is widely recognized that Rab27a plays a critical role in regulating exosome release. Thus, we measured the RAB27A protein level in interscapular BAT of mice after cold exposure and found that RAB27A expression was significantly elevated in response to cold exposure (Supplemental Figure 3A). Consequently, we established *Rab27a* global KO mice (*Rab27a-*KO) and confirmed successful ablation of RAB27A protein expression (Supplemental Figure 3B). BATexos isolated from *Rab27a-*KO mouse BAT showed markedly reduced levels of the exosome hallmark proteins CD63 and TSG101, confirming a defect in exosome biogenesis or secretion upon *Rab27a* loss (Supplemental Figure 3C). Meanwhile, our analysis showed impaired angiogenesis in wounds of *Rab27a-*KO mice, evidenced by decreased CD31 immunostaining (Supplemental Figure 3D). Consistent with this result, *Rab27a-*KO mice exhibited impaired wound healing (Supplemental Figure 3, E and F). Angiogenesis-related genes such as *Vegf* and *Pecam1* were also decreased in *Rab27a-*KO mice (Supplemental Figure 3G). Collectively, these results provide compelling evidence that BAT-derived exosomes play an essential role in promoting endothelial regeneration and accelerating wound repair.

### BATexos promote EC migration and tube formation in vitro.

**Figure 3:**
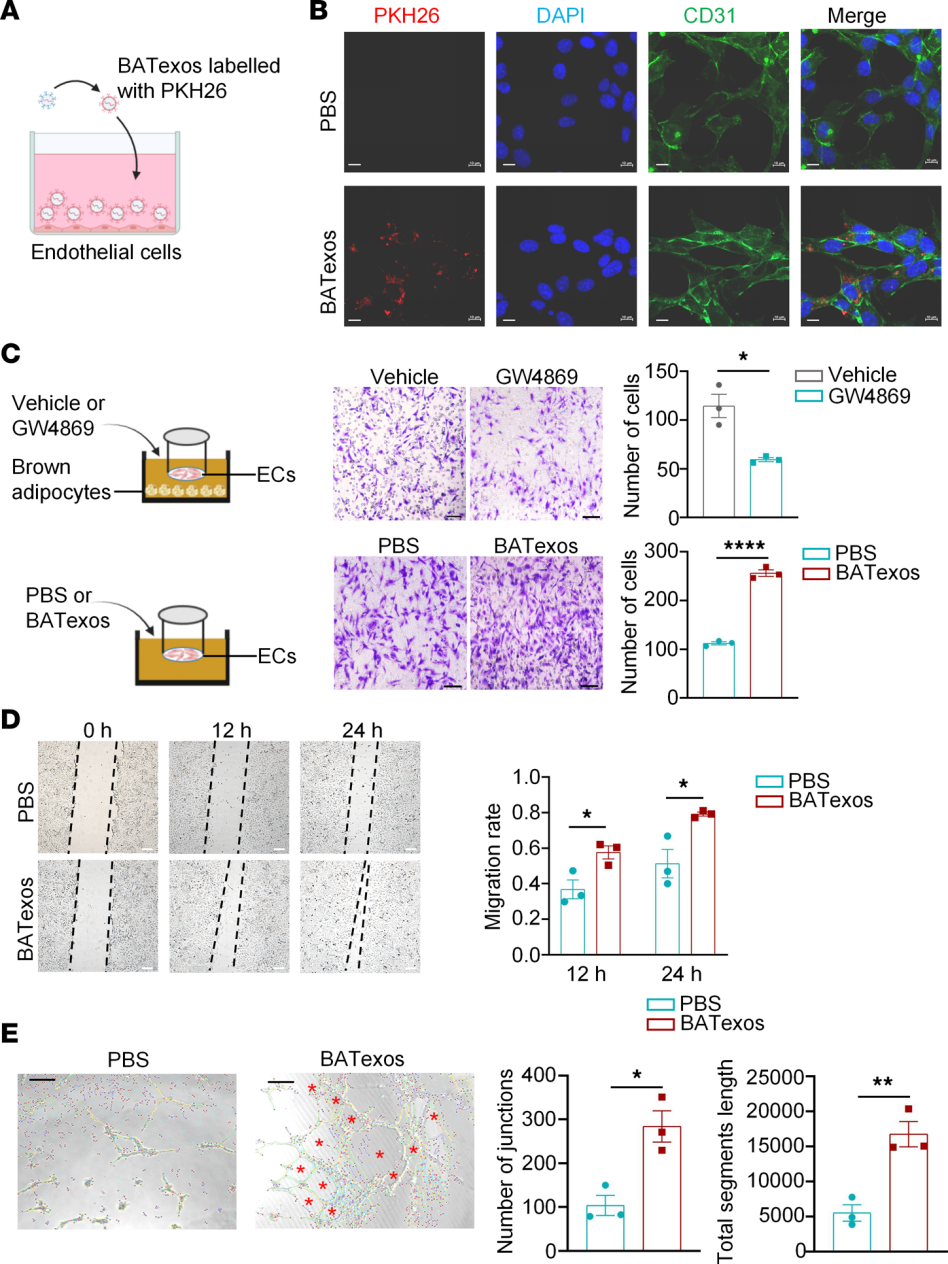
BATexos promote EC migration and tube formation in vitro. (**A**) Schematic of the internalization of exosomes labeled with PKH26 into ECs. (**B**) Representative immunofluorescence images showing the uptake of PKH26-labeled exosomes (red) by CD31^+^ ECs (green). Scale bar: 10 μm. (**C**) Transwell assay and calculation of the effect of GW4869-mediated inhibition of brown adipocyte exosome release or BATexos on EC migration (*n* = 3). Scale bar: 100 μm. (**D**) Scratch assay of ECs treated with BATexos; the migration rate is shown on the right (*n* = 3). Scale bar: 500 μm. (**E**) Endothelial tube formation in response to BATexos treatment (*n* = 3); quantification of the number of junctions and total segment length are shown on the right. Scale bar: 200 μm. The value *n* represents the number of biologically independent samples, from which all experimental data were obtained. Statistical significance was assessed by 2-tailed Student’s *t* test. The data are expressed as mean ± SEM. *P < 0.05, **P < 0.01, ****P < 0.0001.

To determine whether BATexos are internalized by ECs, we labeled BATexos with the red fluorescent lipid dye PKH26 and incubated them with ECs for 24 hours (Figure 3A). Immunofluorescence staining for the endothelial marker CD31 (green) and subsequent confocal microscopy revealed distinct red fluorescent signals (PKH26-BATexos) localized within the cytoplasm of the CD31-positive (green) ECs. This result demonstrated the effective internalization of BATexos by ECs (Figure 3B). To examine the functional effects of BATexos on EC migration, we cocultured brown adipocytes with ECs, as shown in Figure 3C. Inhibition of exosome secretion from adipocytes using GW4869 markedly reduced EC migration, as demonstrated by crystal violet staining (Figure 3C). Meanwhile, we applied isolated BATexos to ECs and found that the EC migration was strongly increased after BATexos treatment compared with the control group (Figure 3C). We also performed a scratch assay in ECs after BATexos treatment to further validate the role of BATexos in EC migration. The scratch-wound assay demonstrated that BATexos-treated ECs migrated significantly faster than PBS-treated controls within 24 hours (Figure 3D). Furthermore, tube formation assays revealed that BATexos treatment substantially increased total tube length and the number of branch points (Figure 3E), indicating enhanced angiogenic capacity (18). Taken together, these findings reveal that BATexos are readily internalized by ECs and promote EC migration and tube formation in vitro.

### Loss of miR-378 in BAT impairs angiogenesis and delays wound healing.

**Figure 4:**
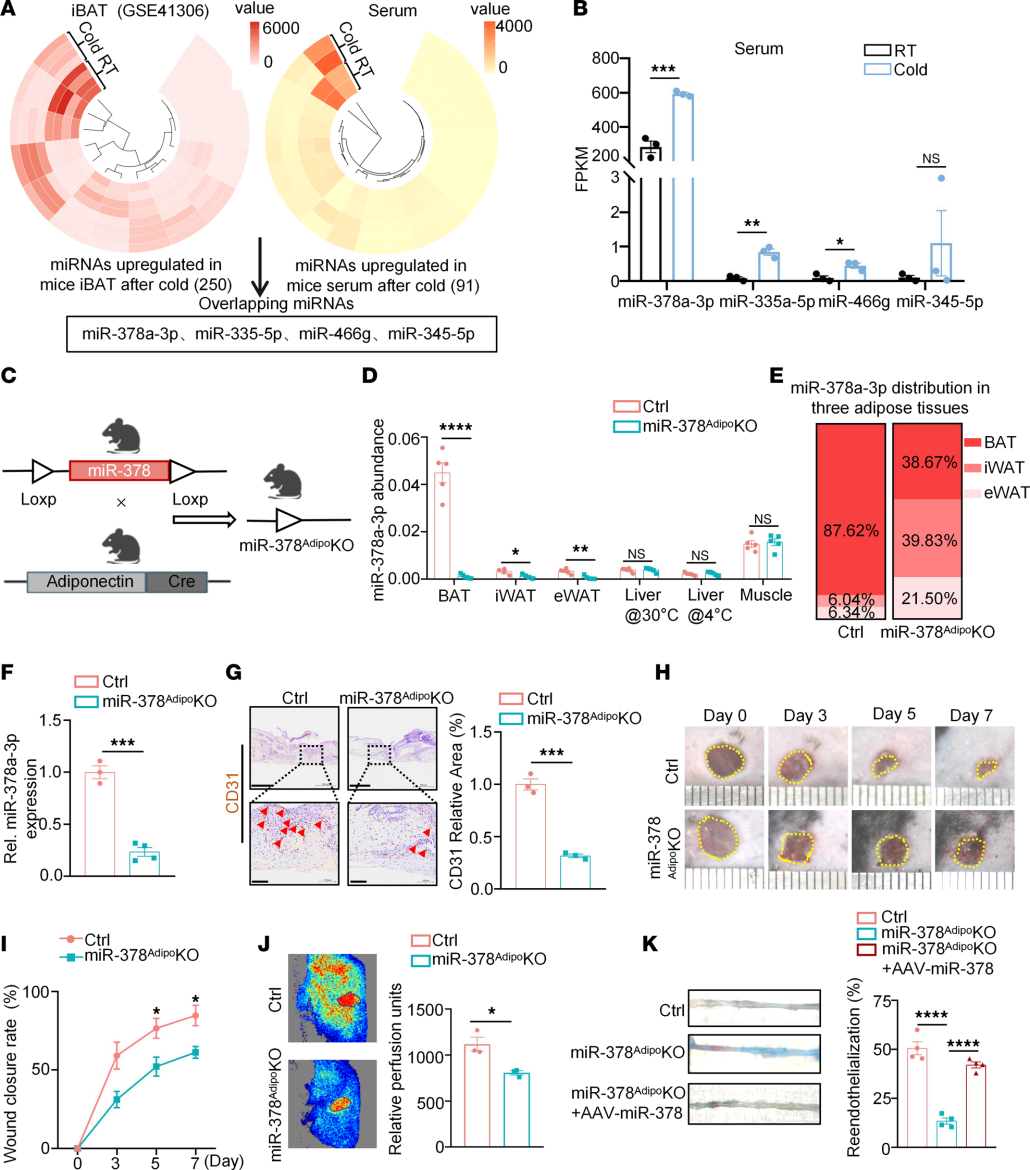
Loss of miR-378 in BAT impairs angiogenesis and delays wound healing. (**A**) Circled heatmap of miRNA-Seq from BAT (GSE41306, left) and serum (right). Serum was pooled from 5 mice per group after 3-day cold exposure. (**B**) Expression levels of miRNA candidates in serum of mice kept at room temperature and 4°C (*n* = 3). (**C**) Schematic showing creation of miR-378^Adipo^ KO mice. (**D**) miR-378a-3p expression levels in BAT, inguinal and epididymal WAT, liver, and muscle of control and miR-378^Adipo^-KO mice (*n* = 4–6). (**E**) Bar chart illustrates relative abundance of miR-378 across different adipose depots, including BAT and inguinal and epididymal WAT. (**F**) qPCR was used to validate miR-378a-3p level in serum of miR-378^Adipo–^KO mice (*n* = 3–4). (**G**) CD31 staining of wounds from the miR-378^Adipo^-KO and littermate control mice. Quantification of CD31 relative area is shown on the right (*n* = 3). Scale bar: 500 μm (top) and 100 μm (bottom). (**H**) Representative images of wounds of control and miR-378^Adipo^-KO mice. (**I**) Quantitative analysis of the wound closure rate of control and miR-378^Adipo^-KO mice (*n* = 3). (**J**) Representative images of blood flow at the wounds in miR-378^Adipo^-KO mice and control mice. The calculated wound microvascular perfusion is shown on the right (*n* = 3). (**K**) Representative images of Evans blue staining of injured femoral artery in control mice, miR-378^Adipo^-KO mice, and AAV-miR-378–treated miR-378^Adipo^-KO mice (*n* = 4). Quantification data of percentage of reendothelialization are shown on the right. The value *n* represents the number of biologically independent samples, from which all experimental data were obtained. Statistical analysis was performed using 2-tailed Student’s *t* test (**B**, **D**, **F**, **G**, **I**, and **J**) or 1-way ANOVA with Tukey’s multiple-comparison test (**K**). The data are expressed as mean ± SEM. NS = not significant, *P < 0.05, **P < 0.01, ***P < 0.001, ****P < 0.0001.

BATexos carry numerous miRNAs that regulate distant organs or tissues (16, 19, 20). To identify the active miRNAs in BATexos involved in promoting angiogenesis and wound healing, we analyzed publicly available microarray data (GSE41306) from NCBI’s Gene Expression Omnibus (GEO), which revealed robust upregulation of several miRNAs in interscapular BAT under cold stimulation (Figure 4A). We subsequently conducted miRNA-Seq of serum from cold-exposed mice and identified a subset of miRNAs that were induced in serum under cold stimulation. Among them, miR-378a-3p, miR-335-5p, miR-446g, and miR-345-5p were significantly upregulated in both serum and interscapular BAT of mice after cold exposure (Figure 4A). Comparative quantification of these 4 candidates in serum showed that miR-378a-3p exhibited the highest expression (Figure 4B). Therefore, we focused primarily on miR-378a-3p in subsequent experiments. To assess the function of miR-378a-3p on vascular repair and wound healing, we created adipose tissue–specific miR-378-KO mice (miR-378^Adipo^-KO) (Figure 4C). In miR-378^Adipo^-KO mice, miR-378a-3p expression was significantly reduced in all adipose depots, including BAT and inguinal and epididymal WAT (Figure 4D). In contrast, its expression in the skeletal muscle and liver at either thermoneutral or cold temperatures remained unchanged, demonstrating a selective deletion in adipose tissue (Figure 4D). In the control mice, BAT was the primary source of miR-378a-3p, contributing to 87.62% of its total miR-378a-3p pool in adipose tissue (Figure 4E). In miR-378^Adipo^-KO mice, miR-378a-3p in BAT showed the greatest reduction (Figure 4, D and E). In addition, circulating levels of miR-378a-3p were markedly reduced in miR-378^Adipo^-KO mice (Figure 4F), suggesting that BAT serves as a major source of circulating miR-378a-3p.

We then sought to establish the link between BAT and systemic/wound miR-378a levels. First, we measured circulating miR-378a-3p in the serum of BATectomized mice and sham-operated mice and found it was significantly reduced after BAT removal (Supplemental Figure 4A). Extending this analysis to the wound tissue itself, we observed a concomitant decrease in both miR-378a-3p and miR-378a-5p levels in BATectomized mice compared with sham controls (Supplemental Figure 4B). Notably, the absolute level of miR-378a-5p in wounds was considerably lower than that of miR-378a-3p (Supplemental Figure 4B). To more directly investigate the role of BATexos, we administered the exosome secretion inhibitor GW4869 by local injection into the BAT of mice. This intervention led to a marked drop in serum miR-378a-3p levels (Supplemental Figure 4C). Consistent with these in vivo findings, direct profiling of isolated BAT-derived exosomes revealed a markedly higher content of miR-378a-3p compared with miR-378a-5p (Supplemental Figure 4D). Collectively, these data provide converging lines of evidence that BAT is a primary source of miR-378a-3p, which is efficiently packaged into exosomes for systemic delivery.

To elucidate the pro-angiogenic role of miR-378a-3p loss in BAT on wound healing, we generated full-thickness dorsal skin wounds in miR-378^Adipo^-KO mice. These mice exhibited reduced CD31 staining at the wound site, indicating impaired angiogenesis (Figure 4G), along with delayed wound closure compared with controls (Figure 4, H and I). Consistently, a reduced microvascular perfusion was observed in miR-378^Adipo^-KO mice (Figure 4J). Moreover, compared with the control mice, the miR-378^Adipo^-KO mice displayed decreased reendothelialization by Evans blue staining (Figure 4K). To investigate the specific role of miR-378a-3p in vascular repair, we performed a rescue experiment in a femoral artery injury model using miR-378^Adipo^-KO mice. Systemic delivery of an adeno-associated virus vector (AAV9-mmu-miR-378a-3p) via tail vein injection significantly restored the impaired reendothelialization observed in miR-378^Adipo^-KO mice. This result indicates that miR-378a-3p is essential for promoting vascular repair (Figure 4K). Similarly, CD31 staining was more intense in the wound tissue of AAV-miR-378–treated mice compared with that in control mice (Supplemental Figure 5A). Consistently, an accelerated wound healing rate was observed in mice treated with AAV relative to control mice (Supplemental Figure 5, B and C). Of note, the level of AKT phosphorylation and PI3K phosphorylation was significantly higher in the wounds of AAV-miR-378–treated mice than in those of control mice (Supplemental Figure 5D). To further validate the crucial role of miR-378a-3p in wound healing, we treated *Rab27a-*KO mice with miR-378^Adipo^-KO BATexos. We found control BATexos enhanced angiogenesis and promoted wound repair of *Rab27a-*KO mice, whereas miR-378^Adipo^-KO BATexos had no similar effect (Supplemental Figure 5, E–G). These results demonstrated that adipose-derived miR-378a-3p, predominantly from BAT, is essential for maintaining vascular integrity and promoting efficient wound healing. Loss of miR-378 in BAT impairs angiogenesis and reendothelialization, while exogenous overexpression enhances vascular repair.

### miR-378a-3p mediates the pro-angiogenic effect of BATexos on ECs.

**Figure 5:**
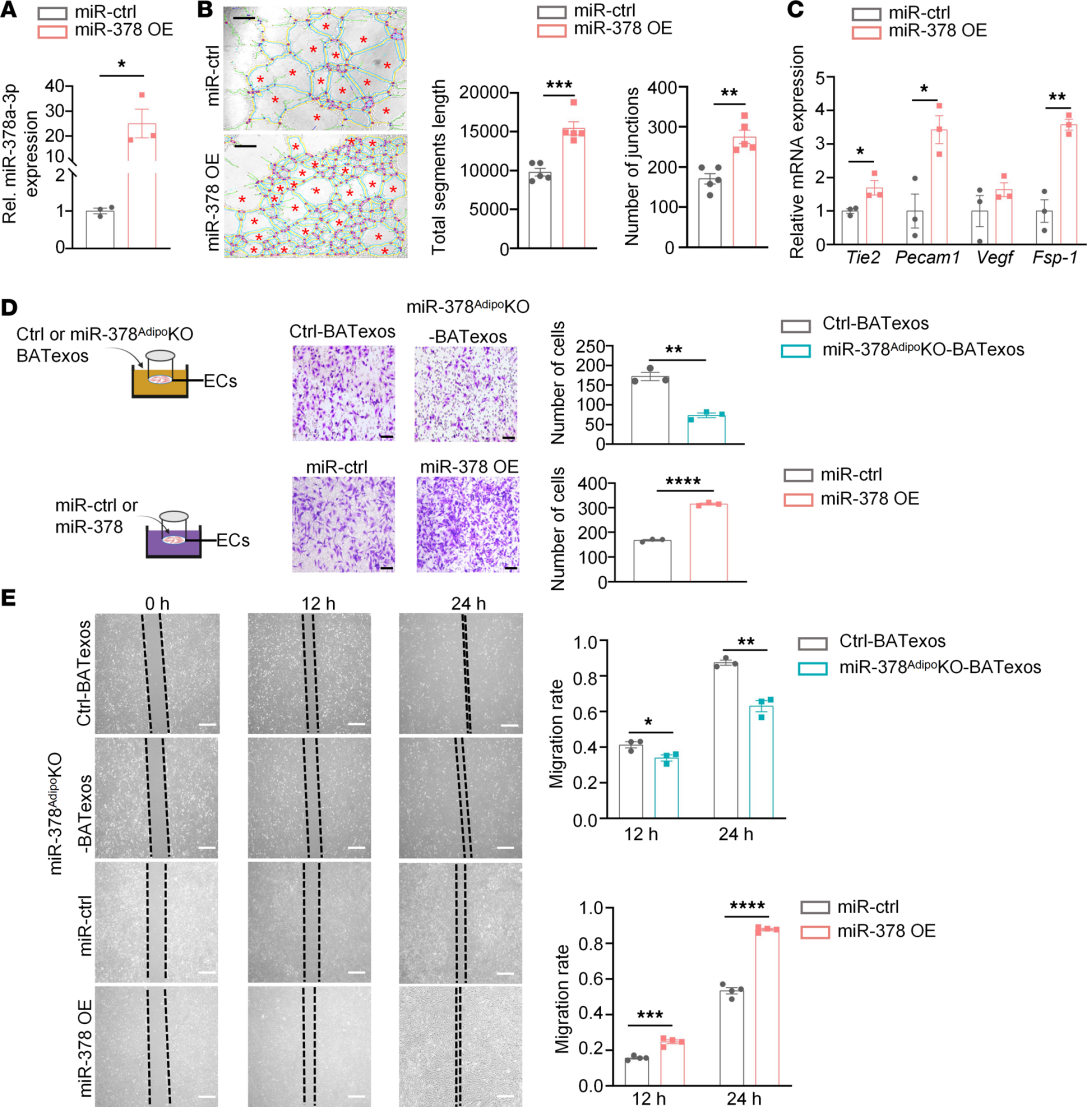
miR-378a-3p mediates the pro-angiogenic effect of BATexos on endothelial cells. (**A**) qPCR was used to measure the miR-378a-3p level in ECs overexpressing miR-378a-3p (*n* = 3). (**B**) Left: representative images of the tube formation assay in ECs overexpressing miR-378a-3p (*n* = 5). Scale bar: 200 μm. Right: quantification of the number of junctions and total segment length. (**C**) Relative expression levels of angiogenesis-related genes in ECs after miR-378a-3p overexpression (*n* = 3). (**D**) Transwell experiment and quantitative analysis of ECs overexpressing miR-378a-3p or cells cocultured with BATexos from miR-378^Adipo^-KO mice or littermate control mice (*n* = 3). Scale bar: 100 μm. (**E**) Scratch assays were used to assess the migration of ECs overexpressing miR-378a-3p or cells cocultured with BATexos from miR-378^Adipo^-KO mice (*n* = 3–4). Scale bar: 500 μm. Quantification of migration rate is shown on the right. The value *n* represents the number of biologically independent samples, from which all experimental data were obtained. Statistical significance was assessed by 2-tailed Student’s *t* test. The data are expressed as mean ± SEM. *P < 0.05, **P < 0.01, ***P < 0.001, ****P < 0.0001.

To further elucidate the mechanism of miR-378a-3p in modulating endothelial angiogenic function, we overexpressed miR-378a-3p in ECs and performed tube formation assays to assess its role in angiogenesis. The results revealed an increased segment length and junctions of ECs after miR-378a-3p overexpression (Figure 5, A and B). In line with these results, the expression of angiogenesis-related genes (such as *Tie2*, *Pecam1*, and *Fsp1*) in the miR-378a-3p–overexpressed ECs was significantly increased compared with controls (Figure 5C). Moreover, to assess its role in cell migration, we performed Transwell assays. miR-378a-3p overexpression in ECs promoted their migration (Figure 5D). In contrast, BATexos isolated from miR-378^Adipo^-KO mice failed to promote EC migration to the same extent as BATexos from control mice (Figure 5D). Consistently, in scratch wound assays, miR-378a-3p overexpression accelerated EC migration within 24 hours. In contrast, treatment with BATexos from miR-378^Adipo^-KO mice resulted in impaired EC migration compared with treatment with control BATexos (Figure 5E). Collectively, these data revealed that miR-378a-3p is a critical mediator of the pro-angiogenic effects of BATexos, promoting both EC migration and tube formation in vitro.

### miR-378a-3p enhances endothelial angiogenic function by targeting Pten and activating the PI3K/AKT signaling pathway.

**Figure 6:**
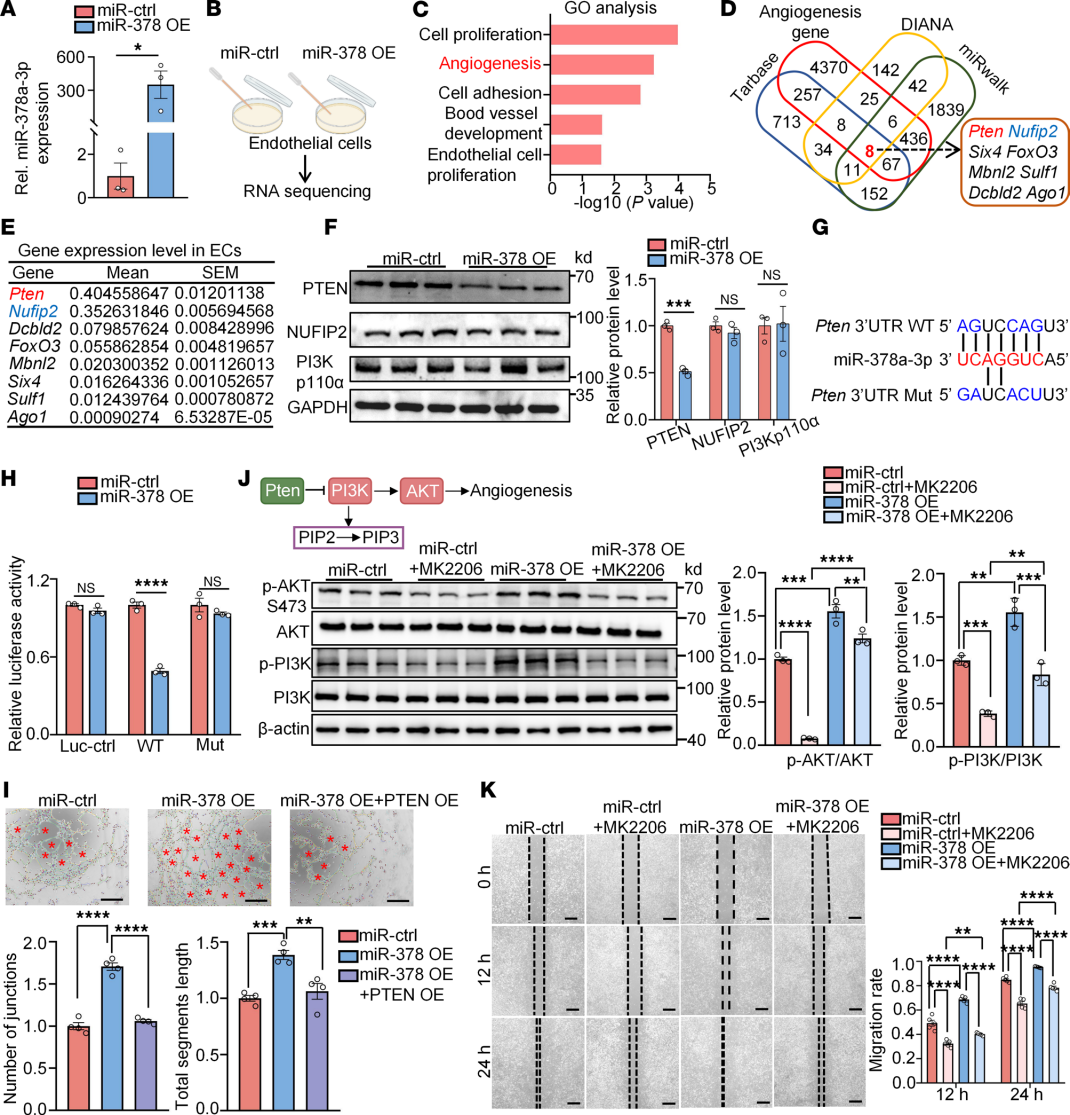
miR-378a-3p enhances endothelial angiogenic function by targeting *Pten* and activating the PI3K/AKT signaling pathway. (**A**) qPCR analysis of miR-378a-3p level (*n* = 3). (**B**) Schematic of RNA-Seq results for ECs overexpressing miR-378a-3p. (**C**) GO analysis of genes upregulated in ECs after overexpressing miR-378a-3p. (**D**) The intersection of the predicted results in the miRWalk database, the DIANA database, the TarBase database, and angiogenesis-related genes. (**E**) qPCR analysis of candidate target genes in ECs. (**F**) Western blot analysis of PTEN, NUFIP2, and PI3K p110α in ECs treated with miR-control and miR-378a-3p (*n* = 3). Right: quantification of the data. (**G**) Schematic illustration of the predicted binding site and corresponding mutation site between the miR-378a-3p and *Pten* 3′UTR. (**H**) Results of dual-luciferase reporter experiments (*n* = 3). (**I**) Representative images of tube formation in ECs transfected with miR-control, miR-378 mimic, or miR-378 mimic plus PTEN overexpression plasmid (*n* = 4). Scale bar: 200 μm. The corresponding quantitative analysis of junction number and tube length is displayed on the bottom. (**J**) Protein levels of p-AKT, AKT, p-PI3K, and PI3K in ECs under 4 conditions: miR-control, miR-378a-3p overexpression, Akt inhibitor (MK2206) treatment, and combined miR-378a-3p overexpression with MK2206 treatment. Quantification of p-AKT/AKT and p-PI3K/PI3K levels in ECs of 4 groups (*n* = 3) (right). (**K**) Scratch assays were used to assess the migration of ECs overexpressing miR-378a-3p or treated with the Akt inhibitor MK2206 (*n* = 5). Scale bar: 500 μm. The corresponding statistical analysis of migration rate is displayed on the right. The value *n* represents the number of biologically independent samples, from which all experimental data were obtained. Statistical analysis was performed using 2-tailed Student’s *t* test (**A**, **F**, and **H**) or 1-way ANOVA with Tukey’s multiple-comparison test (**I**–**K**). The data are expressed as mean ± SEM. NS = not significant, *P < 0.05, **P < 0.01, ***P < 0.001, ****P < 0.0001.

To explore potential mechanisms by which miR-378a-3p promoted EC function, we performed mRNA-Seq analysis in ECs transduced with Lenti-miR-378a (miR-378 OE) compared with the control group (Figure 6, A and B). Gene Ontology (GO) analysis revealed that the angiogenesis-related signaling pathway was enriched after miR-378a-3p overexpression (Figure 6C). To identify direct targets of miR-378a-3p, we examined the intersection of angiogenesis-related genes in the miRWalk, DIANA, and TarBase databases, yielding 8 candidate genes (Figure 6D). Among these, *Pten* and *Nufip2* were highly expressed in these cells (Figure 6E). It has been reported that PI3K p110α is a target gene of miR-378a-3p (17); therefore, we also examined its protein level. Additionally, overexpression of miR-378a-3p in ECs led to a reduction in the protein levels of PTEN but not NUFIP2 and PI3K p110α (Figure 6F); thus, we focused on PTEN for further investigations. Consistently, AAV-miR-378 treatment resulted in a marked decrease in PTEN protein abundance within the wound bed compared with the control group (Supplemental Figure 5D). To confirm direct interaction between miR-378a-3p and *Pten*, we constructed dual-luciferase reporter plasmids containing either WT or mutated *Pten* 3′UTR (Figure 6, G and H). Cotransfection of miR-378a-3p significantly suppressed luciferase activity in the WT construct but had no effect on the mutant, confirming direct binding (Figure 6H). PTEN is known to inhibit angiogenesis through the PI3K/AKT signaling pathway (21). Subsequently, we examined the phosphorylation of AKT and PI3K in ECs after miR-378a-3p overexpression. As expected, overexpression of miR-378a-3p increased phosphorylation levels of both PI3K and AKT in ECs (Figure 6I). Conversely, an Akt inhibitor MK2206 led to inhibition in AKT phosphorylation and PI3K phosphorylation in ECs, which was partially rescued by miR-378a-3p overexpression (Figure 6I). Functionally, overexpression of PTEN attenuated the enhanced tube formation capacity induced by miR-378a-3p (Supplemental Figure 5H and Figure 6J). To further validate the effects of miR-378a-3p and PI3K/AKT signaling on EC migration, we performed a scratch assay: miR-378a-3p overexpression promoted EC migration and Akt inhibition by MK2206 impaired EC migration, which could be partially reversed by miR-378a-3p overexpression (Figure 6K). Together, our findings demonstrated that miR-378a-3p promoted EC migration and tube formation through the PI3K/AKT signaling pathway via suppressing PTEN.

### Liposome-encapsulated miR-378 mimics promote wound healing in diabetic mice.

**Figure 7:**
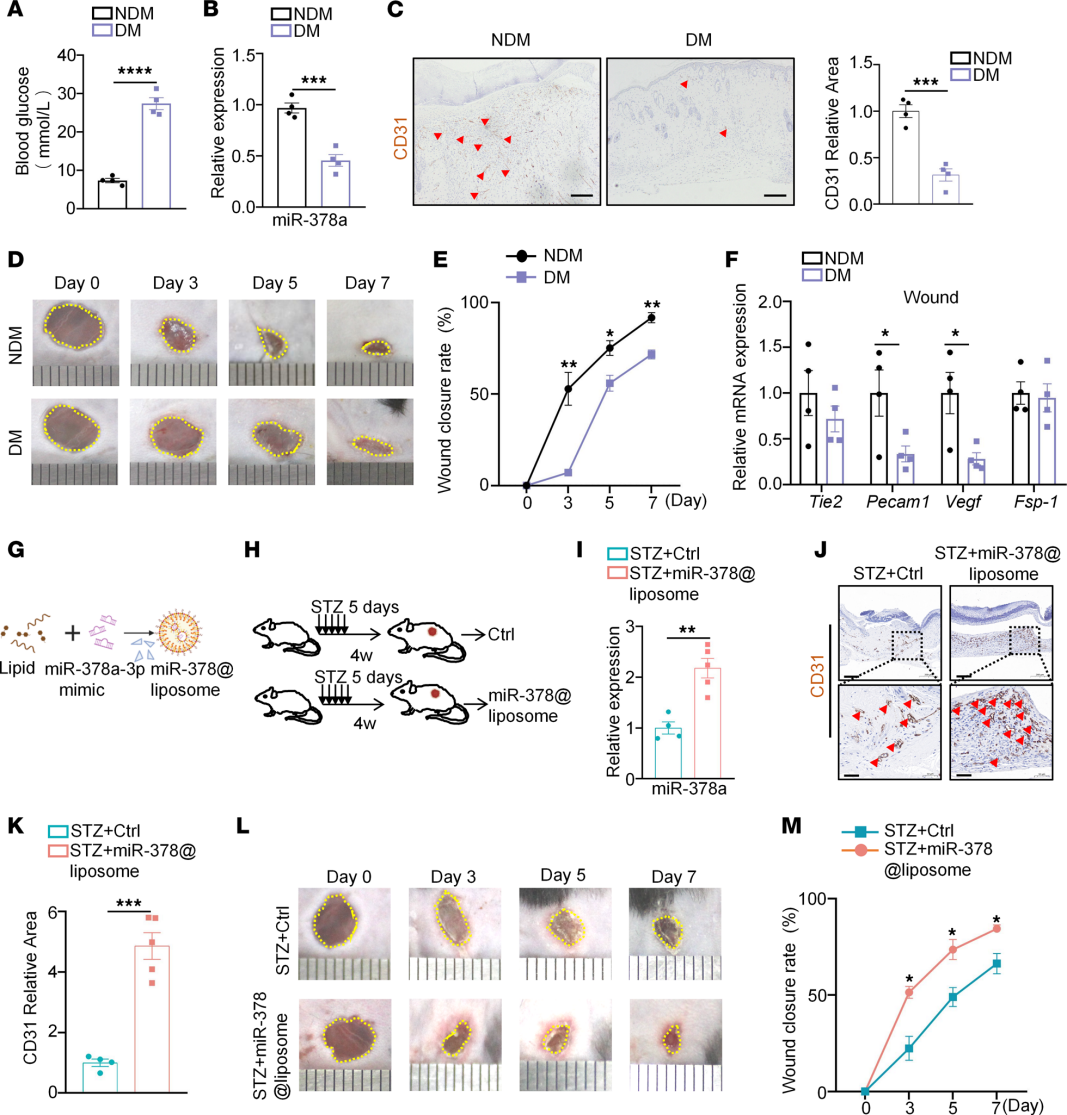
Liposome-encapsulated miR-378 mimics promote wound healing in diabetic mice. (**A**) Random blood glucose in diabetic (DM) and nondiabetic (NDM) mouse wounds. (**B**) Relative expression level of miR-378 in DM and NDM mouse wounds. (**C**) CD31 staining of DM and NDM wounds. Scale bar: 200 μm. Quantification of CD31 relative area is shown on the right (*n* = 4). (**D**) Representative images of DM and NDM wounds. (**E**) Quantitative analysis of wound healing rates in DM and NDM groups. (**F**) Angiogenesis-related genes detected in wounds of DM and NDM mice. (**G**) Schematic illustration of the preparation of miR-378a-3p LNPs. (**H**) Schematic illustration of the DM wound model and treatment with miR-378 LNPs. (**I**) Relative miR-378 levels in DM wounds. (**J**) CD31 staining of DM wounds treated with miR-378 LNPs. Scale bar: 200 μm (top) and 50 μm (bottom). (**K**) Quantification of CD31 relative area in DM wounds treated with control or miR-378 LNPs (*n* = 4–5). (**L**) Representative images of DM wounds treated with miR-378 LNPs (*n* = 3). (**M**) Quantification of wound closure rate in mice treated with miR-378 LNPs (*n* = 3). The value *n* represents the number of biologically independent samples, from which all experimental data were obtained. Statistical significance was assessed by 2-tailed Student’s *t* test. The data are expressed as mean ± SEM. *P < 0.05, **P < 0.01, ***P < 0.001, ****P < 0.0001.

Diabetic wounds are one of the most challenging types of wounds to heal, and there remains an urgent need for effective therapeutic strategies (22). Given the angiogenic potential of miR-378, we hypothesized that it might enhance wound repair in the diabetic setting. Notably, we detected decreased miR-378 expression in mice with diabetes mellitus (DM) compared with those without DM (Figure 7, A and B). Consistent with previous studies, we also found that wound healing was delayed in mice with DM compared with those without DM (Figure 7, C–E). A substantial decrease of angiogenesis-related genes in wounds was observed in mice with DM (Figure 7F). To test whether exogenous delivery of miR-378 could rescue impaired wound healing in diabetes, we generated liposomes encapsulating synthetic miR-378a-3p mimic (miR-378-loaded liposomes) using lipid nanoparticle (LNP) technology, a clinically safe and customizable delivery platform (23–26). We applied these liposomes to the back skin wounds of a diabetic mouse model to explore the effect of miR-378 on diabetic wound healing, which is one of the refractory ulcers (Figure 7, G and H). An elevated expression level of miR-378 was detected in diabetic wounds after the administration of miR-378-loaded liposomes (Figure 7I). Compared with that in the control group, the wound healing rate was higher after miR-378-loaded liposomes treatment, which was consistent with increased CD31 expression in the wound area after miR-378-loaded liposomes treatment, indicating improved angiogenesis (Figure 7, J–M). Taken together, these findings revealed that liposome-based delivery of an miR-378a-3p mimic promotes vascularization and accelerates diabetic wound healing, highlighting its translational potential as a promising therapeutic approach for chronic diabetic ulcer.

## Discussion

The present work sought to identify and characterize BAT-secreted miRNAs that play a key role in wound healing. We found BAT-derived miR-378a-3p accelerated diabetic wound healing and vascular repair by directly targeting PTEN, leading to the activation of PI3K/AKT signaling in ECs. This pathway was characterized using luciferase reporter assays and PTEN rescue experiments. Next, we determined the functional roles of BAT-secreted miR-378a-3p, namely promoting EC migration and tube formation, key steps in angiogenesis. Importantly, this study provides evidence linking BAT-exosomal miR-378a-3p to vascular repair and delineates the mechanism whereby it promotes this process. Furthermore, we demonstrated that the application of miR-378a-3p liposomes to wounds in a diabetic mouse model substantially promoted wound healing.

Diabetic wounds, one of the most challenging diseases to treat, are associated with high disability and mortality rates (22). The present work motivates the pursuit of promising strategies for the treatment of chronic wounds such as diabetic ulcers, ischemic injuries, or age-related impaired healing. In particular, using a BATexos miRNA-based treatment strategy presents 3 key advantages over current therapies: (a) increased precision of miRNA targeted approaches compared with the potential off-target effects of non-specifically modulating growth factors (e.g., VEGF-induced pathological angiogenesis); (b) miRNAs in exosomes are relatively stable and not degraded during transport in circulation (27); (c) reduced risk of immunogenicity associated with treatment relative to administering synthetic nanoparticles (28), as BATexos are naturally occurring biomolecules. Key challenges to developing these therapies include avoiding excessive PI3K/AKT activation, which may increase the risk of tumorigenesis and improving endothelial cell–specific targeting (e.g., exosome surface engineering). Future translational studies will focus on validating our findings in clinical wound specimens. Although the crosstalk between miR-378a-3p, PTEN/PI3K/AKT, and other key developmental pathways is still unclear, substantial progress has already been made in understanding the roles of miRNAs in regulating metabolic homeostasis (29). Specifically, miR-378a-3p has been studied in the context of hepatic gluconeogenesis (17). Studies report that exosomal miRNAs derived from adipose-derived mesenchymal stem cells — notably miR-21, miR-125a-3p, and miR-342-5p — promote endothelial function in the context of angiogenesis (30–32). A recent study showed that miR-378 plays a role in regulating skeletal muscle vascularization (33). Studies reported that miR-378a-3p targeted NF-κB/TNF-α signaling and p110α signaling to promote hepatic inflammation (34) and stimulate hepatic gluconeogenesis (17), respectively. Our work establishes *Pten* as the key target of BATexos miR-378a-3p in ECs, highlighting the context-specificity of miRNA function. Although miR-378a-3p has diverse targets (e.g., PI3K p110α in hepatocytes), our study directly demonstrates that it acts primarily by targeting *Pten* in our system. This cell type–specific targeting thus establishes PTEN as the principal gatekeeper of the PI3K/AKT pathway in the vascular endothelium. Thus, the biological outcome of a circulating exosomal miRNA is determined by the molecular landscape of its recipient cell (35). Our data revealed that miR-378a-3p posttranscriptionally regulates the expression of *Pten*, which encodes a tumor suppressor protein that participates in angiogenesis and negatively regulates the PI3K/AKT signaling pathway (36, 37). *Pten* is a critical tumor suppressor gene whose functional significance extends far beyond vascular ECs, serving as a central regulator across diverse cell types and tissues such as epithelial cells and hepatocytes (38, 39). Although PTEN is widely expressed, its role in vascular ECs is particularly prominent. PTEN orchestrates critical vascular processes, including vascular homeostasis and angiogenesis (40). Notably, we identify a distinct miR-378a-3p/PTEN/PI3K/AKT signaling axis and demonstrate its essential role in endothelial angiogenesis. In light of the potential to leverage other BAT-derived factors as well as additional miR-378a-3p targets for therapeutic benefit, a multiomics approach to investigating adipocyte browning would be of great interest.

An apparent contradiction exists between our finding that miR-378a-3p promotes healing and a prior report that its inhibition accelerates repair (41). This discrepancy may reflect key methodological and biological differences. First, the studies engaged distinct primary cellular targets: Li et al. focused on fibroblast migration, whereas our data demonstrate a pro-angiogenic effect on ECs, suggesting cell context–dependent functions. Secondly, the manipulation strategies differed fundamentally — constitutive transgenic inhibition versus acute, localized mimic supplementation in a KO model — which may engage different compensatory pathways. Finally, variations in delivery systems (nanoparticles vs. liposomes) could affect cellular uptake and local bioavailability. Thus, our findings redefine miR-378a’s role in wound repair by demonstrating its pro-healing action via angiogenesis.

### Limitations

We acknowledge that animal models may not fully replicate human vascular pathophysiology. During target gene screening, we found that the mature sequences of human miR-378 (hsa-miR-378) and mouse miR-378 (mmu-miR-378) are identical, which underscores its high degree of conservation across mammals. This means hsa-miR-378 may also bind to *Pten*, making it a promising clinical target for treating angiogenesis-related diseases. Additionally, the long-term safety profile (e.g., fibrosis, oncogenic risks) of our treatment remains unexplored. Its clinical translation remains an important goal for future research and will require validation using optimized delivery vehicles designed for precision medicine.

## Methods

### Sex as a biological variable.

For experiments in animals, only male mice were used based on experimental considerations. This approach reduces variability associated with the female estrous cycle, allowing us to focus resources on delineating the core mechanism.

### Animals.

WT C57BL/6J mice were purchased from Beijing Weitong Lihua Bioscience Co., Ltd (#000664). Adipose tissue–specific miR-378a-KO (miR-378^Adipo^-KO) mice and *Rab27a*-KO (*Rab27a-*KO) were generated by Cyagen Biosciences, Inc. The mice were raised in a controlled environment (12-hour light/12-hour dark, 22°C–24°C, 40%–60% humidity) with free access to water and food at a specific pathogen–free barrier facility. The mice were euthanized by CO_2_ inhalation in compliance with institutional guidelines.

### Cell line and cell culture.

The murine cerebral microvascular EC line bEnd.3 was purchased from Wuhan Pricella Biotechnology Co., Ltd. Cell culture was performed in 5% CO_2_ at 37°C in a cell incubator. The cell culture medium consisted of 90% DMEM (11965092, Gibco), 10% FBS (FBSST-01033, OriCell), and 1% penicillin-streptomycin (P1400, Solarbio). For validation of the importance of the PI3K/AKT signaling pathway, ECs were treated with the Akt inhibitor MK2206 (10 μM, HY-10358, MCE). ECs were transfected with a miR-378 mimics (5 nmol, miR1CM001, RiboBio) via Lipofectamine 3000 transfection reagent (L3000008, Invitrogen/Life Technologies) according to the manufacturer’s instructions. Brown stromal vascular fraction (SVF) cells were obtained from 8-week-old C57BL/6 male mice. In brief, BAT was harvested, washed in ice-cold PBS, minced, and digested in DMEM-F12 (11320033, Gibco) containing 1 mg/mL type I collagenase (BS163, Biosharp) for 40 minutes at 37°C. The solution was filtered through a 70 μm cell strainer and centrifuged for 5 minutes at 150*g*. Then, the cell pellet was washed with DMEM-F12 twice and cultured in medium consisting of 90% DMEM-F12, 10% FBS (FBSST-01033, OriCell), and 1% penicillin-streptomycin (P1400, Solarbio). Then, the SVF cells were immortalized via SV40 large T antigen transfection (Obio Technology Corp., Ltd.) according to the manufacturer’s protocol. For brown adipocyte differentiation, confluent SVF cells were treated with DMEM F-12 medium containing 1 nM T3 (T2877, Sigma-Aldrich), 1 μM rosiglitazone (R2408, Sigma-Aldrich), 5 μg/mL insulin (I8040, Solarbio), 0.5 mM 3-isobutyl-1-methylxanthine (I7018, Sigma-Aldrich), 2 μg/mL dexamethasone (D1756, Sigma-Aldrich), and 125 μM indomethacin (I7378, Sigma-Aldrich) for 2 days. The induction medium was subsequently changed to maintenance medium (5 μg/mL insulin, 1 nM T3, and 1 μM rosiglitazone). The maintenance cocktail was changed every 2 days. The HEK293 cell line was obtained from Wuhan Pricella Biotechnology Co., Ltd. HEK293 cells were cultured in medium containing 90% DMEM, 10% FBS, and 1% penicillin-streptomycin.

### Tube formation assay.

For tube formation assays, ECs were seeded equally into 96-well plates precoated with Matrigel (88427045, ABW). Images of tube formation were captured with an inverted phase-contrast microscope after incubation for 6 hours. The tube length and number of junctions were analyzed with ImageJ (NIH) software.

### Transwell assay.

For the Transwell experiment, ECs were digested and resuspended in DMEM without FBS after 24 hours. The cell suspension was added to the top chambers of a Transwell plate. The chambers were collected and fixed after incubation for 24 hours. Cells on the lower membrane surface were fixed and stained with 0.1% crystal violet solution for 20 minutes at room temperature. The crystal violet–stained cells were imaged under a microscope.

### Scratch assay.

ECs were seeded into 6-well plates until they were fully confluent. A sterile 200 μL pipette tip was used to draw a straight line across the plate, which was subsequently washed with PBS. Images were captured at 0 hours, 12 hours, and 24 hours with a microscope. The EC migration rate was measured via ImageJ (NIH) software.

### Lentivirus transfection.

Lenti-miR-378a was constructed and packaged by Genechem Co., Ltd. For cell transfection, 1 × 10^8^ Vg/μL lenti-miR-378a (1 μL) was added to a 12-well plate when the ECs reached 50%–60% confluence.

### Dual-luciferase reporter assay.

The seed region sequences containing the WT and MUT sequences of the *Pten* 3′ UTR were separately cloned and inserted into a luciferase reporter vector. HEK293 cells were cotransfected with 100 ng of the indicated vector constructs and the miR-378 mimic/control (Guangzhou RiboBio Co., Ltd.). HEK293 cells were collected and lysed after 48 hours with a dual-luciferase reporter kit (DL101-01, Vazyme). Then, the cell supernatant was used to assess luciferase activity and Renilla luciferase activity. The firefly luciferase reporter activity was measured and calculated by normalization to Renilla luciferase activity.

### BATexos isolation and treatment.

BATexos were separated by ultracentrifugation. The extraction of exosomes from BAT was performed as follows: BAT was excised from the mice and cut with scissors. The minced tissue was subsequently incubated with type I collagenase for 30 minutes at 37°C. The mixture was subsequently centrifuged at 300*g* at 4°C for 5 minutes after digestion, and the pellets were discarded. The supernatant was collected by sequential centrifugation at 2,000*g* for 10 minutes and 10,000*g* for 30 minutes at 4°C and then centrifuged for 70 minutes at 120,000*g* at 4°C to obtain exosomes. The exosomes were finally dispersed in PBS and stored at –80°C. For tail vein injection, exosomes were collected from adult C57BL/6J mice, and exosomes (100 μg in 100 μL of PBS) were injected into each mouse every 3 days. For local injection into the wound, exosomes (100 μg in 100 μL of PBS) were s.c. injected at 4 sites around the wound.

### GW4869 and CL316,243 injection.

Before the wound model or femoral artery wire injury model was established, 100 μL of GW4869 (3 mg/kg) (D1692, Sigma-Aldrich) was locally administered into the BAT by multipoint injection every 3 days until the mice were euthanized. CL316,243 (C5976, Sigma-Aldrich) was i.p. injected for 3 consecutive days before the wound model was established and for another 4 days (1 mg/kg body weight) after the wound model was established.

### BAT excision.

The mice were anesthetized with isoflurane (R510-22-10, RWD), and a small incision was made on the back. The BAT was exposed, and the vasculature was ligated via silk suture to prevent bleeding. The BAT was then gently dissected, and the incision was carefully closed with surgical sutures. Sham-operated mice underwent all steps except for excision of the BAT. All the mice were allowed to recover for 7 days.

### Transmission electron microscopy.

Twenty microliters of the resuspended exosome samples were added dropwise to 200-mesh grids and incubated at room temperature for 10 minutes. Then, the grids were negatively stained with 2% phosphotungstic acid for 3 minutes, and the remaining liquid was removed with filter paper. The samples were then observed with an HT7800 transmission electron microscope.

### AAV production and administration.

AAV9-mmu-miR-378a was designed, amplified, and purified by Genechem Co., Ltd. A total of 1 × 10^11^ vg/μL of AAV9-mmu-miR-378a (100 μL) was administered via tail vein injection on the day of femoral artery wire injury.

### RNA-Seq.

The mRNA of the ECs was isolated and sent to BGI Genomics Co., Ltd. for mRNA-Seq. Gene expression analysis (differentially expressed gene and Kyoto Encyclopedia of Genes and Genomes pathway enrichment analysis) was further performed by a BGISEQ-500 sequencing platform (MGI Tech Co., Ltd.). High-quality samples (RIN > 8.0, assessed by Agilent Fragment Analyzer) were used for library preparation and subsequent paired-end sequencing on the Illumina NovaSeq 6000 platform at BGI Genomics. Bioinformatic analysis involved aligning clean reads to the *GRCm39* mouse genome with Bowtie2, quantifying transcripts via RNA-Seq by Expectation-Maximization, and identifying differentially expressed genes with DESeq2 (FDR-adjusted *P* < 0.05 and |log_2_FC| > 1). For miRNA expression analysis, each sample was an independent pooled serum sample prepared from 8-week-old male C57BL/6J mice. Serum miRNAs were enriched using the miRNeasy Serum/Plasma kit (QIAGEN, 217184). Briefly, 200 μL of serum was mixed with QIAzol lysis reagent, followed by chloroform phase separation. The aqueous phase was recovered, and ethanol was added to bind RNA to the RNeasy MinElute spin column. After washing, total RNA (including small RNAs) was eluted in RNase-free water. RNA quality and concentration were assessed using an Agilent 2100 Bioanalyzer with the Small RNA kit. Libraries were constructed using the NEBNext Multiplex Small RNA Library Prep Set (Illumina), and sequencing was performed on an Illumina NextSeq platform with single-end 75-bp reads.

### RNA isolation and qPCR.

RNAiso Plus (9109, Takara) was used to obtain RNA from cells or tissues. The RNA was reverse-transcribed with a cDNA synthesis kit. Real-time PCR was conducted on a QuantStudio1 system (Applied Biosystems) according to the manufacturer’s instructions. The gene primers used are listed in Supplemental Table 1. For exosome RNA isolation, the extracted exosomes were lysed with RNA-easy Isolation reagent (R701, Vazyme) for 5 minutes at room temperature. The mixture was subsequently centrifuged at 12,000*g* for 15 minutes at room temperature to precipitate impurities. Subsequently, 550 μL of the supernatant was transferred to a new centrifuge tube, and an equal volume of isopropanol was added. The mixture was centrifuged at 12,000*g* for 10 minutes at room temperature to precipitate the RNA after incubation at room temperature for 10 minutes. Then, the precipitate was resuspended with 1 mL of 75% ethanol at 8,000*g* for 3 minutes at room temperature, and the supernatant was discarded. The pellet was air-dried and dissolved in RNase-free ddH_2_O. Exosomal miRNA was reverse-transcribed by using a miRNA 1st Strand cDNA Synthesis kit (by stem-loop) (MR101-01, Vazyme).

### Western blotting.

Briefly, protein was extracted from cells and tissues with NP40 lysis buffer (B1027, BIO4U, MCE) supplemented with a proteinase inhibitor cocktail (HY-K0010, MCE) and phosphatase inhibitor cocktails (HY-K0021 and HY-K0022, MCE). Equal amounts of protein were separated on SDS-PAGE, and the proteins were subsequently transferred to PVDF membranes. The PVDF membranes were subsequently blocked with 5% nonfat milk for 1 hour at room temperature. After being washed with TBST, the PVDF membranes were incubated overnight with the following primary antibodies: anti-CD31 (1:1,000, 77699, Cell Signaling Technology); anti-UCP1 (1:1,000, U6382, Sigma-Aldrich); anti-β-actin (1:1,000, 20536-1, Proteintech); anti-CD63 (1:1,000, 10628D, Invitrogen); anti-TSG101 (1:1,000, 23296, Invitrogen); anti-RAB27A (1:1,000, 17817-1, Proteintech); anti-PI3K p110α (1:1,000, 4249, Cell Signaling Technology); anti-GAPDH (1:2000, 10494-1, Proteintech); anti-NUFIP2 (1:1,000, 17752-1, Proteintech); anti-AKT (1:1,000, 4691, Cell Signaling Technology); anti-p-AKT (1:1,000, 66644-1, Proteintech); anti-PI3K (1:1,000, 4257, Cell Signaling Technology); anti-p-PI3K (1:1,000, AF3241, Affinity), and anti-PTEN (1:1,000, 9559, Cell Signaling Technology). Afterward, the membranes were incubated with a secondary antibody for 1 hour. ECL developing solution was applied to the membranes for exposure with an imaging system.

### Femoral artery wire-induced injury animal model.

WT male C57BL/6 mice were subjected to left femoral artery wire injury. Briefly, isoflurane was applied to anesthetize the animals. The left femoral artery was dissected after the tissues were separated. Sutures were applied proximally and distally to control vascular flow. A small incision was made in the femoral artery, after which a 0.014” guidewire was inserted into the common femoral artery. The incision was maintained for 2 minutes to achieve hemostasis. Subsequently, the guide wire was removed to prevent bleeding from the arteriotomy. At 14 days after injury, the mice were anesthetized and euthanized, and the injured arterial segments were collected.

### Mouse femoral artery endothelial denudation and reendothelialization.

Anesthetized mice were injected with 200 μL Evans blue dye (2%) through the tail vein at 7 days after femoral artery injury. Five minutes later, mice were perfused with PBS. The injured artery was collected and photographed with a stereomicroscope. To measure the reendothelialized area, we analyzed reendothelialization with Image-Pro Plus software. The blue-stained area represented the area not repaired with ECs. The reendothelialization rate was calculated as the percentage of the non–blue-stained area over the initially injured area.

### Mouse dorsal skin wound model.

Mouse hair was removed with depilatory cream and disinfected with 70% ethanol. After anesthesia, a hole punch (8 mm) was applied to remove the skin on the back, and a full-thickness skin defect model was created. For the diabetic wound model, WT male C57BL/6 mice received an i.p. injection of streptozotocin (50 mg/kg) (S0130, Sigma-Aldrich) for 5 consecutive days. Blood glucose levels were tested 1 week after injection, and hyperglycemia was defined as a blood glucose level greater than 11.1 mmol/L. After 4 weeks, these hyperglycemic mice were used to generate wounds on the back.

### Laser speckle contrast imaging.

Cutaneous blood flow was assessed non-invasively using a Laser Speckle Contrast Imager (RWD Life Science Co., Ltd, RFLSI-ZW). Anesthetized mice were placed on a heating pad (37°C) in a prone position. The wound area was kept unobstructed. Speckle contrast images were captured at a working distance of 20 cm under consistent low ambient light. Perfusion images were generated in real-time by the system software RFLSI Analysis (version 02.02.05.39826), which calculates the speckle contrast value inversely related to blood flow velocity. To quantify perfusion, a standardized region of interest (ROI) encompassing the entire wound area was defined. The mean perfusion intensity (in arbitrary perfusion units, PU) within the ROI was recorded. The data were expressed in PU.

### Preparation of liposomes.

In brief, C12-200 (HY-145405, MCE), cholesterol (HY-N0322, MCE), 1,2-distearoyl-sn-glycero-3-phosphocholine (DSPC) (HY-W040193, MCE), and methoxypolyethylene glycol-1,2-dimyristoyl-rac-glycerol (mPEG-DMG) (112764, MCE) were dissolved in ethanol at a molar ratio of 50:38.5:10:1.5. miR-378 mimics were dissolved in diethyl pyrocarbonate (DEPC) water. The lipids and the strand-specific, double-stranded miR-378a-3p mimic oligonucleotide (miR10003151-1-5, Guangzhou RiboBio Co., Ltd.) were then rapidly vortexed to ensure full mixing. Ultracentrifugation (120,000*g*, 70 minutes, 4°C) was used to separate the uncaptured mmu-miR-378a-3p mimic. The obtained liposomes loaded with mmu-miR-378a-3p mimic were diluted in PBS.

### Metabolic analysis in mice.

Oxygen consumption, food intake, and respiratory exchange ratio were measured for 48 hours in metabolic cages. The mice were acclimated to their individual housing and calorimetry chamber for 24 hours and kept at a room temperature of approximately 24°C under a 12-hour light/12-hour dark cycle. For the glucose tolerance test, mice were fasted for 16 hours and i.p. injected with a glucose solution in saline (1.5 g/kg). Blood glucose levels were measured at 0 minutes, 15 minutes, 30 minutes, 60 minutes, 90 minutes, and 120 minutes after injection.

### ELISA.

The assay was performed strictly according to the manufacturer’s protocol (SEB345Mu, Cloud clone). Briefly, mice serum samples (clarified by centrifugation at 3,000*g* for 15 minutes) were prepared. Then, 100 μL of standard or diluted sample was added to the antibody-precoated wells and incubated at 37°C for 1 hour. After incubation, the liquid was discarded, and 100 μL of Detection Antibody A working solution was added to each well, followed by another 1-hour incubation at 37°C. The plate was subsequently washed 3 times. After discarding the wash buffer, 100 μL of HRP-conjugated Detection Antibody B working solution was added to each well and incubated at 37°C for 30 minutes. After another 5 washes, 90 μL of 3,3’,5,5’-tetramethylbenzidine (TMB) substrate solution was added to each well for color development at 37°C in the dark for 10–20 minutes. The reaction was then stopped by adding 50 μL of stop solution per well, and the OD of each well was immediately measured at 450 nm using a microplate reader. The concentration of CD63 was calculated based on a 4-parameter logistic standard curve.

### Histology analysis.

For H&E staining, BAT tissues were fixed in 4% paraformaldehyde, embedded in paraffin, and cut into 5 μm sections. Sections were then stained by H&E. For IHC, BAT, wounds, and femoral arteries were fixed in 4% paraformaldehyde overnight, dehydrated, and embedded in paraffin for sectioning. Sections were probed with antibodies for UCP1 (1:1,000, ab234430, Abcam) and CD31 (1:100, 77699, Cell Signaling Technology) for 2 hours at room temperature and washed 3 times with PBS. Then, the sections were incubated with HRP-conjugated secondary antibodies (1:200, GB23303, Servicebio) for 1 hour at room temperature, followed by 3,3′-diaminobenzidine development, dehydration, and mounting.

### Immunofluorescence.

ECs cultured on glass slides were fixed in 4% paraformaldehyde for 15 minutes. After washing 3 times with PBS, cells were permeabilized with 0.1% Triton X-100 in PBS for 10 minutes and blocked in 5% BSA for 30 minutes at room temperature. Cells were then stained with primary antibodies against CD31 (1:200, 5585, Cell Signaling Technology) overnight at 4°C. Cells were washed and incubated with secondary antibodies for 1 hour at room temperature. DAPI was used to visualize the nuclei. Images were taken by LSM900 Airyscan confocal microscope.

### Statistics.

All statistical analyses were carried out using GraphPad Prism version 8.0. The data are displayed as the mean ± SEM. The significance of differences among groups was determined with 2-tailed *t* tests or 1-way ANOVA. Differences with a *P* value less than 0.05 were regarded as statistically significant.

### Study approval.

All experiments involving animals were conducted according to the ethical policies and procedures approved by the IACUC of Tongji Hospital, Tongji Medical College, Huazhong University of Science and Technology, Wuhan, China (approval TJH-202104008).

### Data availability.

The data that support the findings of this study are available in the main text or the supplemental materials; values for all data points in graphs are reported in the Supporting Data Values file. RNA-Seq data and miRNA-Seq data have been deposited in NCBI’s BioProject (PRJNA1406089 and PRJNA1440143).

## Author contributions

YC conceived and supervised the study. HD designed and performed most experiments. YX contributed to manuscript revision and data analysis. JL performed the cell experiments. JG assisted in the establishment of animal models. QK, RH, ZW, XP, ZZ, WW, YL, RG, and RP assisted in sample collection. HD and MY wrote the draft of the manuscript. YC edited and revised the manuscript. HD was listed as the first co–first author because of their contribution to experiments; YX was listed second because of their contribution to manuscript revision and data analysis; JL was listed third because of their contribution to cell experiments; and JG was listed fourth because of their work with animal models. All authors read and approved the manuscript.

## Conflict of interest

The authors have declared that no conflict of interest exists.

## Funding support

The following organizations provided funding support:

- National Natural Science Foundation of China grant 82270910 (to YC).
- National Natural Science Foundation of China grant 82570997 (to YC).
- National Natural Science Foundation of China grant 82350610277 (to YC).
- National Natural Science Foundation of China grant 82070859 (to YC).
- National Key R&D Program of China grant 2022YFA0806100 (to YC).

## Supplementary Material

Supplemental data

Unedited blot and gel images

Supporting data values

## Version 1 04/21/2026

In-Press Preview

## Version 2 06/08/2026

Electronic publication
