# Supplementary material for: BAT-derived miR-378a-3p facilitates endothelial angiogenic function and promotes wound healing
Source: JCI Insight. 2026 Apr 21;11(11):e201311. doi: 10.1172/jci.insight.201311 (PMC13313551; doi:10.1172/jci.insight.201311)
Supplement: Supplemental data [file jciinsight-11-201311-s297.pdf]

Supplementary Figure legends:

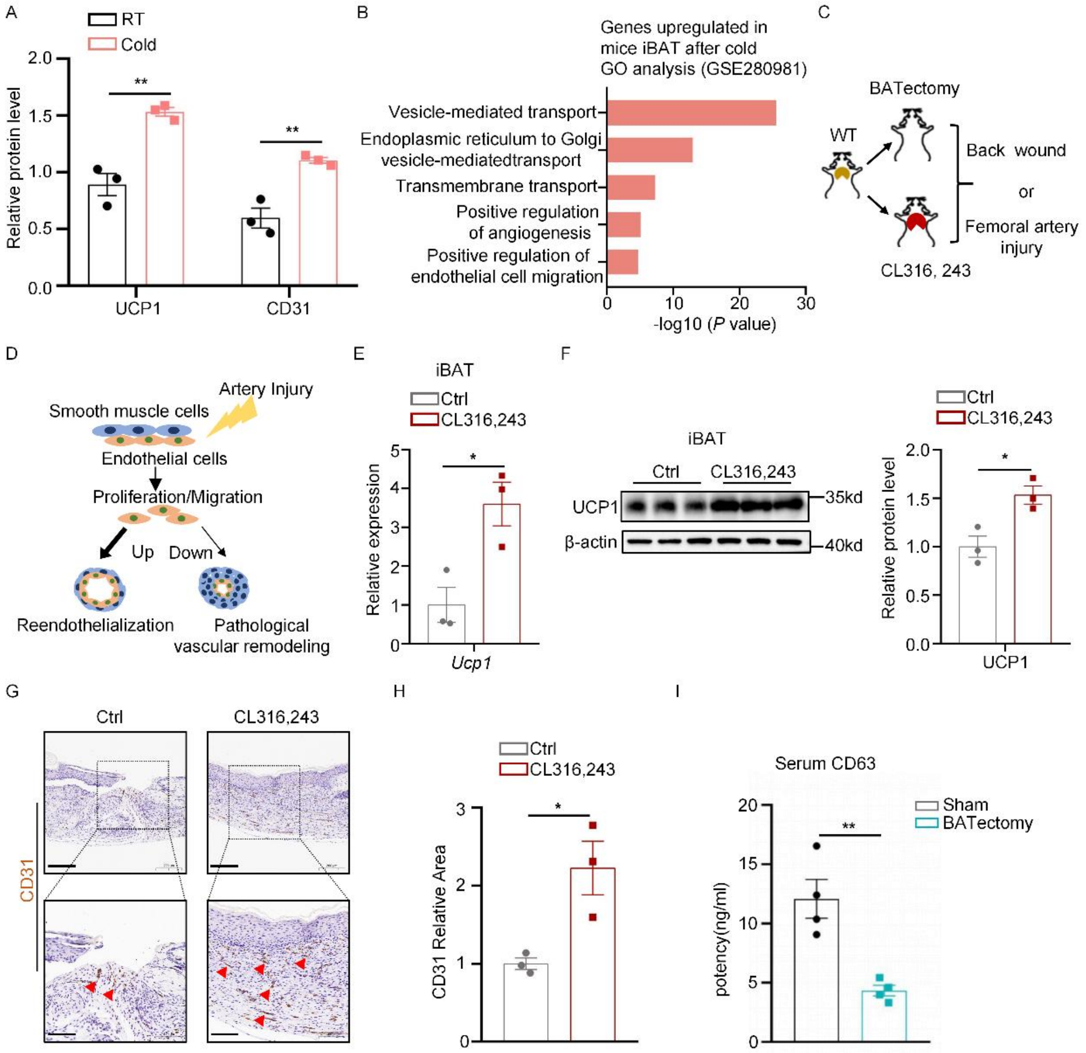

Figure S1

Supplementary Figure 1. CL316,243 injection into brown adipose tissue (BAT) significantly promotes angiogenesis.

(A) Quantitative analysis of UCP1 and CD31 protein levels normalized to β-actin in BAT from mice subjected to cold exposure. (B) Gene Ontology analysis of cold-induced upregulated genes in iBAT (GSE280981). (C) Schematic diagram of the wound model and guidewire-induced femoral artery injury with BATectomy or CL-316,243 injection. (D) Schematic diagram illustrating the role of ECs in vascular repair. (E) Relative mRNA level of *Ucp1* in BAT from mice treated with CL316,243. (F) Relative protein level of UCP1 in BAT from mice treated with CL316,243. Quantification of protein levels normalized to β-actin was shown right. (G) CD31 staining of wound tissue from mice treated with CL316,243. Scale bar: 200 μm (top) and 100 μm (bottom). (H) Quantification of CD31 Relative area in wounds from mice treated with CL316,243. (I) Effect of BAT ablation on serum CD63 concentration. The value n represents the number of biologically independent samples, from which all experimental data were obtained. Statistical significance was assessed by two-tailed Student's t-test. The data are expressed as the mean ± SEM. n.s. = not significant, *$P < 0.05$, **$P < 0.01$, ***$P < 0.001$, ****$P < 0.0001$.

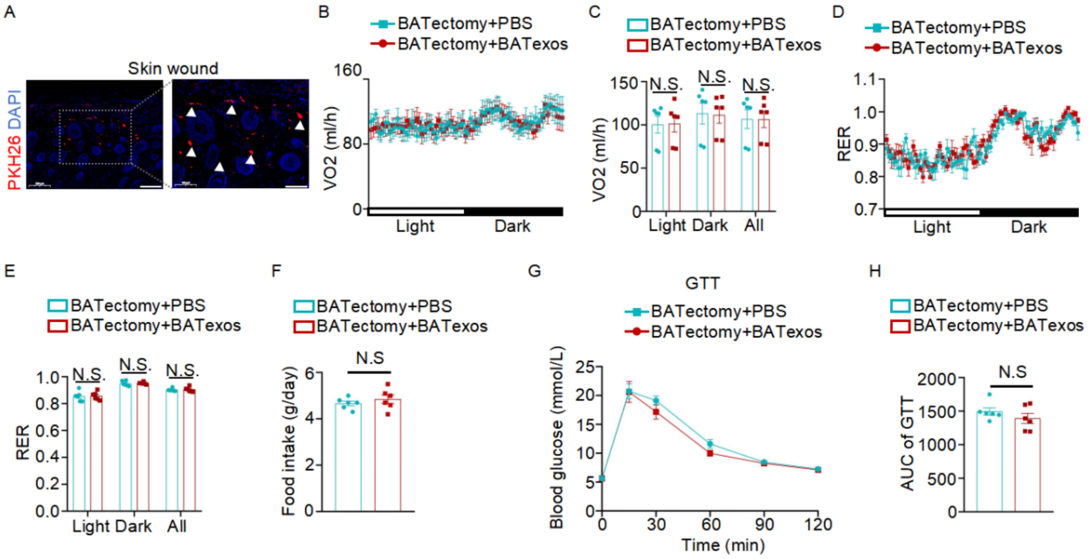

A
Skin wound
PKH26 DAPI
B
BATectomy+PBS
BATectomy+BATexos
160
80
0
VO2 (ml/h)
Light
Dark
C
BATectomy+PBS
BATectomy+BATexos
N.S.
N.S.
N.S.
150
100
50
0
VO2 (ml/h)
Light Dark All
D
BATectomy+PBS
BATectomy+BATexos
1.1
1.0
0.9
0.8
0.7
RER
Light
Dark
E
BATectomy+PBS
BATectomy+BATexos
N.S.
N.S.
N.S.
1.0
0.8
0.6
0.4
0.2
0.0
RER
Light Dark All
F
BATectomy+PBS
BATectomy+BATexos
N.S
6
4
2
0
Food intake (g/day)
G
GTT
BATectomy+PBS
BATectomy+BATexos
25
20
15
10
5
0
Blood glucose (mmol/L)
0 30 60 90 120
Time (min)
H
BATectomy+PBS
BATectomy+BATexos
N.S
2000
1500
1000
500
0
AUC of GTT

Figure S2

Supplementary Figure 2. BATexos promote angiogenesis without altering energy metabolism.

(A) Immunofluorescence staining for PKH26-labeled BATexos (red) in wound tissue. Scale bar: 200 μm (left) and 100 μm (right). (B-C) Oxygen consumption (B) and its quantitative analysis (C) of BAT-ablated mice treated with the BATexos (n = 6). (D-E) Respiratory exchange ratio (RER) (D) and quantitative analysis (E) of BAT-ablated mice treated with the BATexos (n = 6). (F) Food intake of BAT-ablated mice treated with the BATexos (n = 6). (G) Glucose tolerance test of BAT-ablated mice treated with the BATexos. (H) Area under the curve (AUC) quantification of the glucose tolerance test results (n = 6). The value n represents the number of biologically independent samples, from which all experimental data were obtained. Statistical significance was assessed by two-tailed Student's t-test. The data are expressed as the mean ± SEM. n.s. = not significant, *$P < 0.05$, **$P < 0.01$, ***$P < 0.001$, ****$P < 0.0001$.

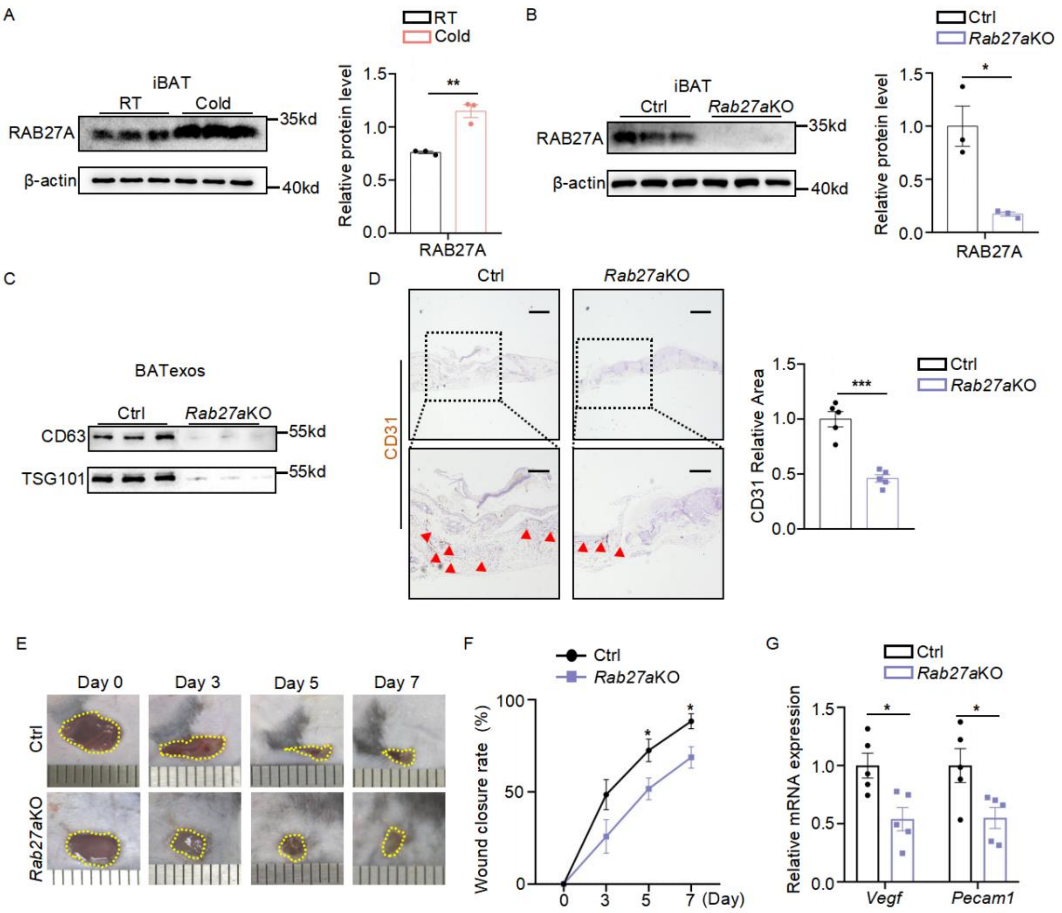

A
RT
Cold
iBAT
RT
Cold
RAB27A
35kd
β-actin
40kd
**
Relative protein level
RAB27A
B
Ctrl
Rab27aKO
iBAT
Ctrl
Rab27aKO
RAB27A
35kd
β-actin
40kd
*
Relative protein level
RAB27A
C
BATexos
Ctrl
Rab27aKO
CD63
55kd
TSG101
55kd
D
Ctrl
Rab27aKO
CD31
***
CD31 Relative Area
E
Day 0
Day 3
Day 5
Day 7
Ctrl
Rab27aKO
F
Ctrl
Rab27aKO
Wound closure rate (%)
(Day)
G
Ctrl
Rab27aKO
Relative mRNA expression
Vegf
Pecam1

Figure S3

Supplementary Figure 3. Inhibiting release of exosomes impairs vascular repair and wound healing.

(A) Western blot analysis of RAB27A in the iBAT of mice exposed to RT or cold. The right panel showed the quantification. (B) Validation of RAB27A protein knockout in the iBAT of control and *Rab27a*KO mice. Quantification of RAB27A protein was shown right. (C) Western blot analysis of exosome specific marker proteins (CD63 and TSG101) in the BATexos from ctrl mice and *Rab27a*KO mice. (D) CD31 staining of wounds in ctrl mice and *Rab27a*KO mice. Scale bar: 500 μm (top) and 200 μm (bottom). Quantitative analysis of CD31-positive area in the wound bed was shown right. (E) Representative images of wounds from control mice and *Rab27a*KO mice ($n = 5$). (F) Quantification of wound closure rate in ctrl mice and *Rab27a*KO mice ($n = 5$). (G) *Vegf* and *Pecam1* mRNA levels in ctrl mice and *Rab27a*KO mice ($n = 5$). The value n represents the number of biologically independent samples, from which all experimental data were obtained. Statistical significance was assessed by two-tailed Student's t-test. The data are expressed as the mean ± SEM. n.s. = not significant, $*P < 0.05$, $**P < 0.01$, $***P < 0.001$, $****P < 0.0001$.

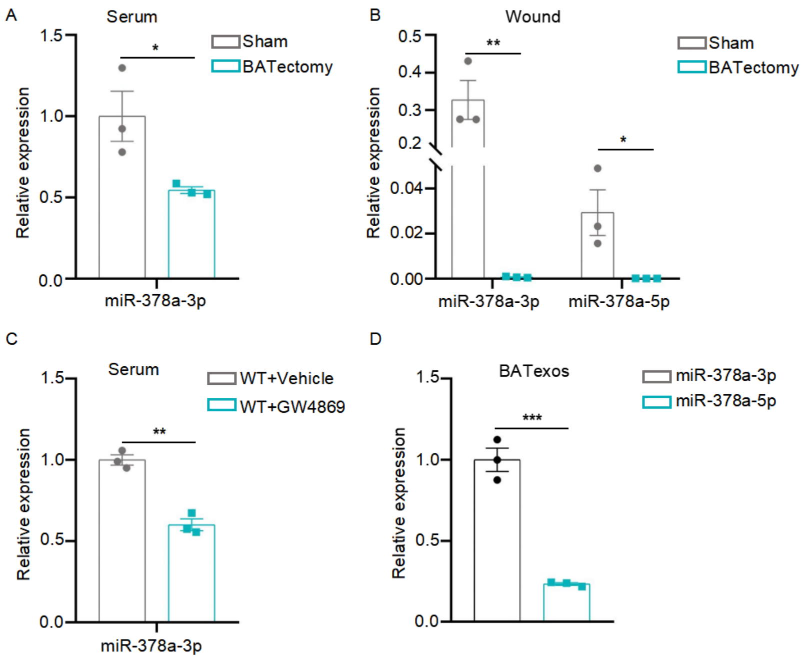

A
Serum
1.5
1.0
0.5
0.0
Relative expression
*
Sham
BATectomy
miR-378a-3p
B
Wound
0.5
0.4
0.3
0.2
0.04
0.02
0.00
Relative expression
**
*
Sham
BATectomy
miR-378a-3p
miR-378a-5p
C
Serum
1.5
1.0
0.5
0.0
Relative expression
**
WT+Vehicle
WT+GW4869
miR-378a-3p
D
BATexos
1.5
1.0
0.5
0.0
Relative expression
***
miR-378a-3p
miR-378a-5p

Figure S4

Supplementary Figure 4. Differential expression of miR-378 across multiple tissues in response to experimental interventions.

(A) Serum miR-378a-3p expression was quantified by qPCR in sham-operated mice (Sham, n = 3) and BAT-ablated mice (BATectomy, n=3). (B) Relative expression level of miR-378a-3p and miR-378a-5p in the wounds from in sham-operated mice and BATectomy mice (n = 3). (C) The expression of miR-378a-3p was evaluated in WT mice treated with vehicle or GW4869 (n = 3). (D) The expression level of miR-378a-3p and miR-378a-5p in BATexos (n = 3). The value n represents the number of biologically independent samples, from which all experimental data were obtained. Statistical significance was assessed by two-tailed Student's t-test. The data are expressed as the mean ± SEM. n.s. = not significant, *$P < 0.05$, **$P < 0.01$, ***$P < 0.001$, ****$P < 0.0001$.

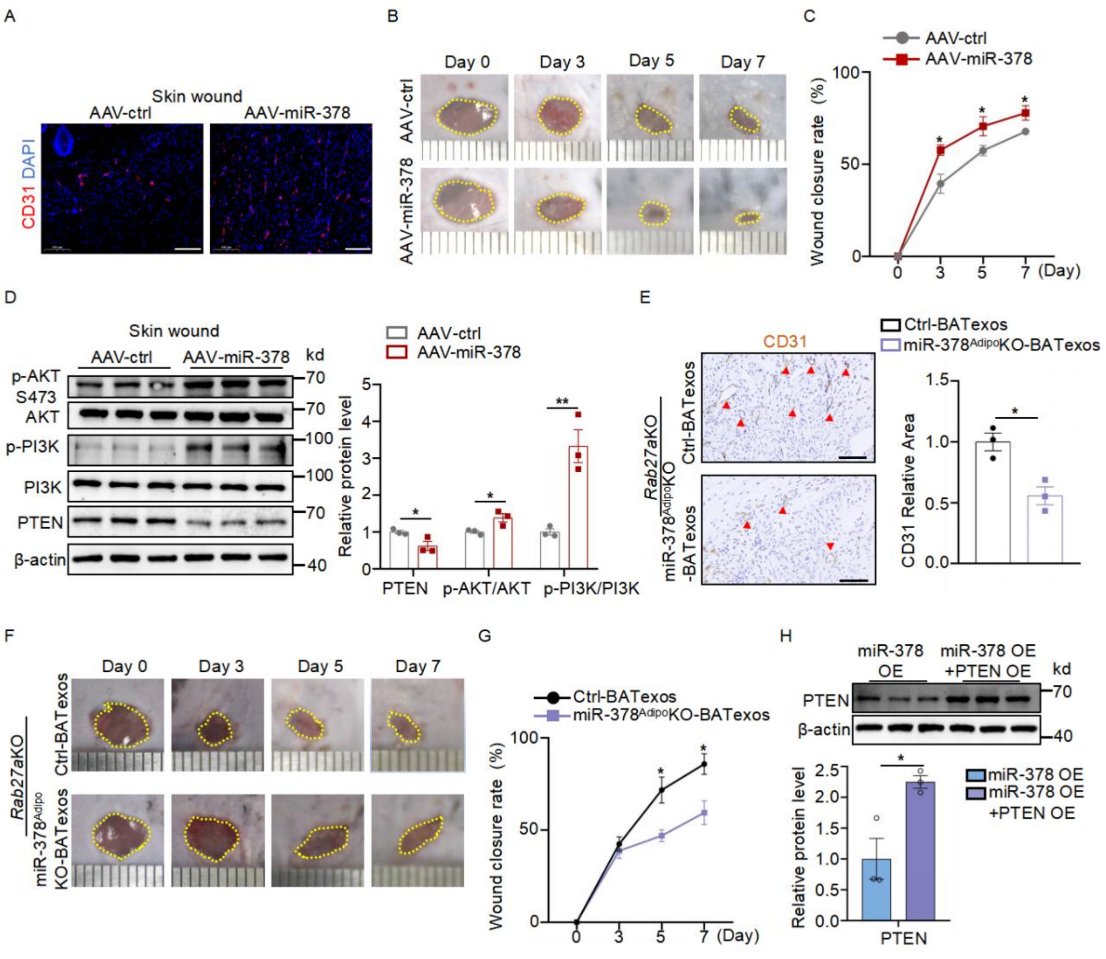

A
Skin wound
AAV-ctrl
AAV-miR-378
CD31 DAPI
B
Day 0
Day 3
Day 5
Day 7
AAV-ctrl
AAV-miR-378
C
AAV-ctrl
AAV-miR-378
Wound closure rate (%)
(Day)
D
Skin wound
AAV-ctrl
AAV-miR-378
kd
p-AKT S473
AKT
p-PI3K
PI3K
PTEN
β-actin
Relative protein level
PTEN
p-AKT/AKT
p-PI3K/PI3K
E
CD31
Ctrl-BATexos
miR-378AdipoKO-BATexos
Rab27aKO
miR-378AdipoKO -BATexos
CD31 Relative Area
F
Rab27aKO
Ctrl-BATexos
miR-378Adipo KO-BATexos
G
Ctrl-BATexos
miR-378AdipoKO-BATexos
Wound closure rate (%)
(Day)
H
miR-378 OE
miR-378 OE +PTEN OE
kd
PTEN
β-actin
Relative protein level
PTEN

Figure S5

Supplementary Figure 5. miR-378 promotes vascular repair and wound healing.

(A) Immunofluorescence staining for CD31(red), a marker for ECs, and DAPI (blue) for nuclei in wounds of mice treated with AAV-ctrl or AAV-miR-378.Scale bar, 100 μm. (B) Representative images of wounds in mice treated with AAV-ctrl or AAV-miR-378. (C) Quantification of wound closure rate in mice treated with AAV-ctrl or AAV-miR-378 (n = 6). (D) Western blot analysis of the protein levels of PTEN, p-AKT, AKT, p-PI3K, and PI3K in skin wounds from mice treated with AAV-ctrl or AAV-miR-378. Quantification of protein levels was shown right (n = 3). (E) CD31 staining of wounds in *Rab27a*KO mice treated with Ctrl-BATexos or miR-378^{Adipo}KO-BATexos. Quantitative analysis of CD31-positive area in the wound bed was shown right (n = 3). Scale bar, 100 μm. (F) Representative images of wounds in *Rab27a*KO mice treated with miR-378^{Adipo}KO-BATexos. (G) Quantification of wound closure rate in *Rab27a*KO mice treated with miR-378^{Adipo}KO-BATexos (n = 3). (H) The protein level of PTEN in ECs treated with miR-378 mimic and miR-378 mimic + PTEN overexpression plasmid. Quantitative data are presented in the bottom panel (n = 3). The value n represents the number of biologically independent samples, from which all experimental data were obtained. Statistical significance was assessed by two-tailed Student's t-test.The data are expressed as the mean ± SEM. n.s. = not significant, *$P < 0.05$, **$P < 0.01$, ***$P < 0.001$, ****$P < 0.0001$.

Graphic Abstract: Schematic diagram of the regulation of BAT-derived miR-378a-3p in endothelial cells during wound healing.

Under cold exposure, BAT-derived miR-378a-3p is delivered to endothelial cells, where it triggers a signaling cascade: suppression of PTEN followed by activation of the PI3K/AKT pathway. This cascade drives key angiogenic events—enhanced endothelial migration and tube formation—thereby accelerating wound healing. Figure created using BioRender (https://biorender.com).

49 Spadina Ave. Suite 200
Toronto ON M5V 2J1 Canada
www.biorender.com

Confirmation of Publication and Licensing Rights - Open Access

March 19th, 2026

Subscription Type:	*Student Plan - Academic*
Agreement number:	*OL29HP9358*
Publisher Name:	*JCI insight*
Figure Title:	*Schematic diagram of the regulation of BAT-derived miR-378a-3p in endothelial cells during wound healing.*
Citation to Use:	*Created in BioRender. Chen, Y. (2026) https://BioRender.com/4egb240*

To whom this may concern,

This document ("Confirmation") hereby confirms that Science Suite Inc. dba BioRender ("BioRender") has granted the following BioRender user: Yong Chen ("User") a BioRender Academic Publication License in accordance with BioRender's Terms of Service and Academic License Terms ("License Terms") to permit such User to do the following on the condition that all requirements in this Confirmation are met:

1) publish their Completed Graphics created in the BioRender Services containing both User Content and BioRender Content (as both are defined in the License Terms) in publications (journals, textbooks, websites, etc.); and
2) sublicense such Completed Graphics under "open access" publication sublicensing models such as CC-BY 4.0 and more restrictive models, so long as the conditions set forth herein are fully met.

Requirements of User:

1) All Completed Graphics to be published in any publication (journals, textbooks, websites, etc.) must be accompanied by the following citation either as a caption, footnote or reference for each figure that includes a Completed Graphic:
"Created in BioRender. Chen, Y. (2026) https://BioRender.com/4egb240 ".
2) All terms of the License Terms including all Prohibited Uses are fully complied with. E.g. For Academic License Users, no commercial uses (beyond publication in journals, textbooks or websites) are permitted without obtaining or switching to a BioRender Industry Plan.
3) A Reader (defined below) may request that the User allow their figure to be a public template for Readers to view, copy, and modify the figure. It is up to the User to determine what level of access to grant.

Open-Access Journal Readers:

Open-Access journal readers ("Reader") who wish to view and/or re-use a particular Completed Graphic in an Open-Access journal subject to CC-BY sublicensing may do so by clicking on the URL link in the applicable citation for the subject Completed Graphic.

The re-use/modification options below are available after the Reader requests the User to adapt their figure as a BioRender template and the User has granted such access.

1) View-Only/Free Plan Use: A Reader who wishes to only view the Completed Graphic may do so in the BioRender Services as either a BioRender Free Plan user or simply as a viewer. By becoming a BioRender Free Plan user, the Reader may view, modify and re-use the Completed Graphic as permitted under BioRender's Basic License Terms (e.g. personal use only, no publishing or commercial use permitted).
2) Re-Use/Publish with No Modifications: For any re-use and re-publication of a Completed Graphic with no modification(s) to the Completed Graphic made by the Reader, a Reader may do so by citing the original author using the citation noted above with the Completed Graphic. The Reader must also comply with the underlying License Terms which apply to the Completed Graphic as noted above (e.g. no commercial use for Academic License).
3) Re-Use/Publish with Modifications: For any re-use and re-publication of a Completed Graphic with a modification(s) made by the Reader, the Reader may do so by becoming a BioRender user themselves under either an Academic or Industry Plan, citing the original author using the citation noted above with the Completed Graphic and complying with the applicable License Terms.

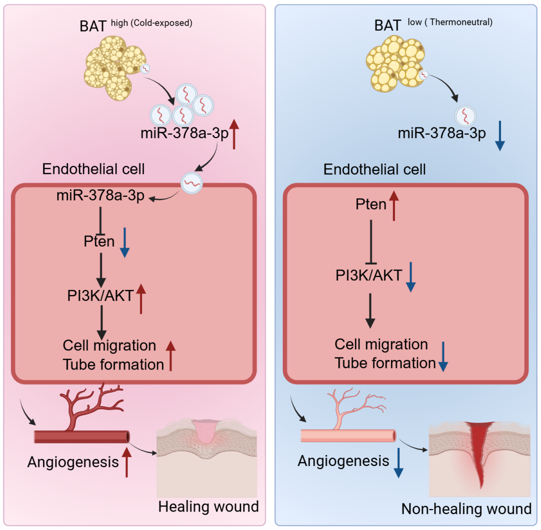

For any questions regarding this document, or other questions about publishing with BioRender, please refer to our BioRender Publication Guide, or contact BioRender Support at support@biorender.com.

49 Spadina Ave. Suite 200
Toronto ON M5V 2J1 Canada
www.biorender.com

Confirmation of Publication and Licensing Rights - Open Access

March 20th, 2026

Subscription Type: *Student Plan - Academic*
Agreement number: *AT29HUI820*
Publisher Name: *JCI insight*

Figure Title: *Schematic diagram*

Citation to Use: *Created in BioRender. Chen, Y. (2026) https://BioRender.com/5c57wh6*

To whom this may concern,

This document ("Confirmation") hereby confirms that Science Suite Inc. dba BioRender ("BioRender") has granted the following BioRender user: Yong Chen ("User") a BioRender Academic Publication License in accordance with BioRender's Terms of Service and Academic License Terms ("License Terms") to permit such User to do the following on the condition that all requirements in this Confirmation are met:

1) publish their Completed Graphics created in the BioRender Services containing both User Content and BioRender Content (as both are defined in the License Terms) in publications (journals, textbooks, websites, etc.); and
2) sublicense such Completed Graphics under "open access" publication sublicensing models such as CC-BY 4.0 and more restrictive models, so long as the conditions set forth herein are fully met.

Requirements of User:

1) All Completed Graphics to be published in any publication (journals, textbooks, websites, etc.) must be accompanied by the following citation either as a caption, footnote or reference for each figure that includes a Completed Graphic:
"Created in BioRender. Chen, Y. (2026) https://BioRender.com/5c57wh6 ".
2) All terms of the License Terms including all Prohibited Uses are fully complied with. E.g. For Academic License Users, no commercial uses (beyond publication in journals, textbooks or websites) are permitted without obtaining or switching to a BioRender Industry Plan.
3) A Reader (defined below) may request that the User allow their figure to be a public template for Readers to view, copy, and modify the figure. It is up to the User to determine what level of access to grant.

Open-Access Journal Readers:

Open-Access journal readers ("Reader") who wish to view and/or re-use a particular Completed Graphic in an Open-Access journal subject to CC-BY sublicensing may do so by clicking on the URL link in the applicable citation for the subject Completed Graphic.

The re-use/modification options below are available after the Reader requests the User to adapt their

figure as a BioRender template and the User has granted such access.

1) <u>View-Only/Free Plan Use:</u> A Reader who wishes to only view the Completed Graphic may do so in the BioRender Services as either a BioRender Free Plan user or simply as a viewer. By becoming a BioRender Free Plan user, the Reader may view, modify and re-use the Completed Graphic as permitted under BioRender's Basic License Terms (e.g. personal use only, no publishing or commercial use permitted).
2) <u>Re-Use/Publish with No Modifications:</u> For any re-use and re-publication of a Completed Graphic with no modification(s) to the Completed Graphic made by the Reader, a Reader may do so by citing the original author using the citation noted above with the Completed Graphic. The Reader must also comply with the underlying License Terms which apply to the Completed Graphic as noted above (e.g. no commercial use for Academic License).
3) <u>Re-Use/Publish with Modifications:</u> For any re-use and re-publication of a Completed Graphic with a modification(s) made by the Reader, the Reader may do so by becoming a BioRender user themselves under either an Academic or Industry Plan, citing the original author using the citation noted above with the Completed Graphic and complying with the applicable License Terms.

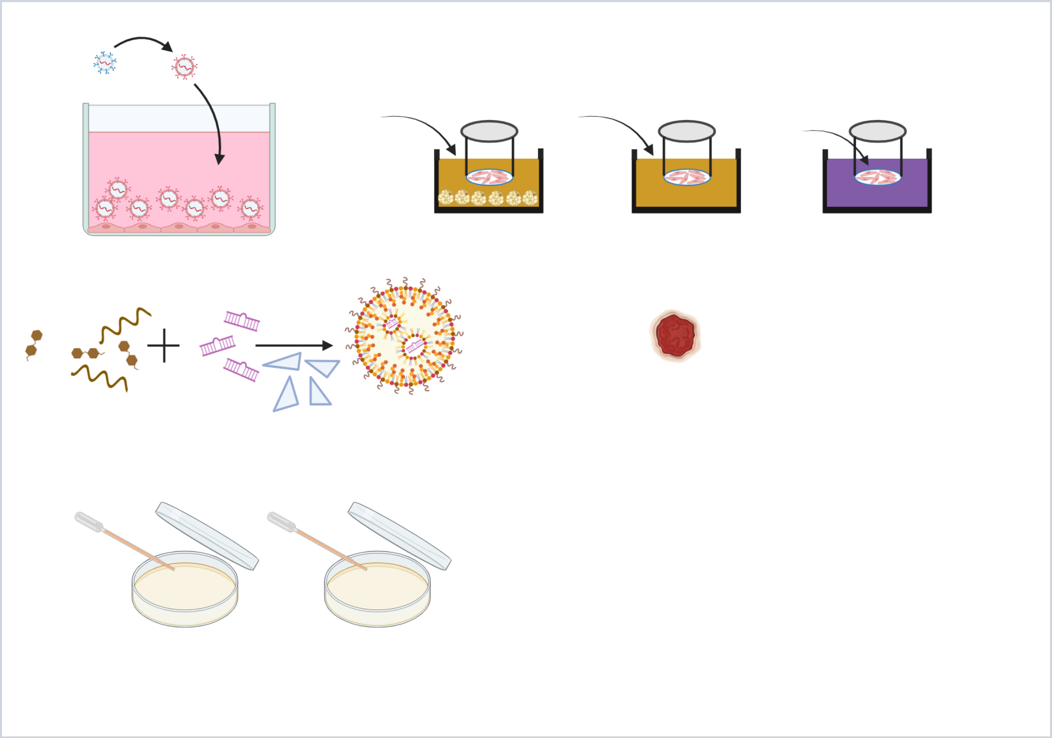

For any questions regarding this document, or other questions about publishing with BioRender, please refer to our BioRender Publication Guide, or contact BioRender Support at support@biorender.com.
