# Supplementary material for: BAT-derived miR-378a-3p facilitates endothelial angiogenic function and promotes wound healing
Source: JCI Insight. 2026 Apr 21;11(11):e201311. doi: 10.1172/jci.insight.201311 (PMC13313551; doi:10.1172/jci.insight.201311)

Full unedited gel for Figure 1C

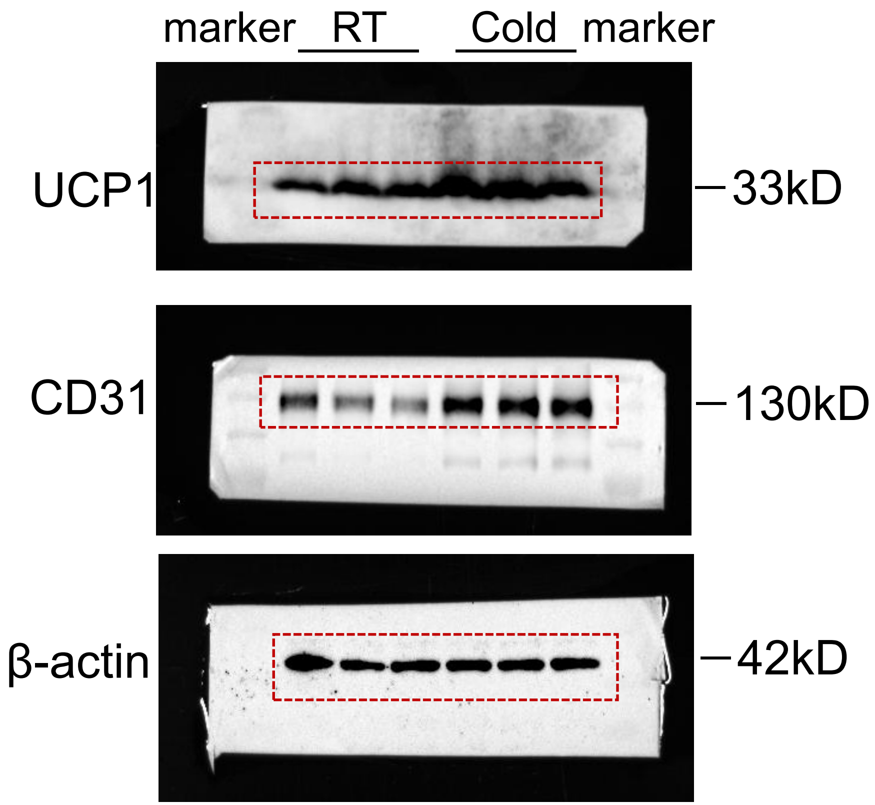

Full unedited gel for Figure 2D

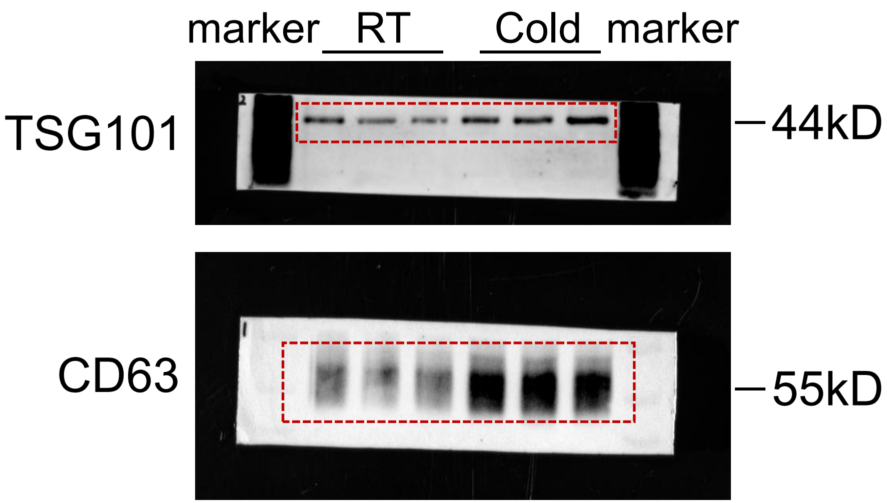

Full unedited gel for Figure 6F

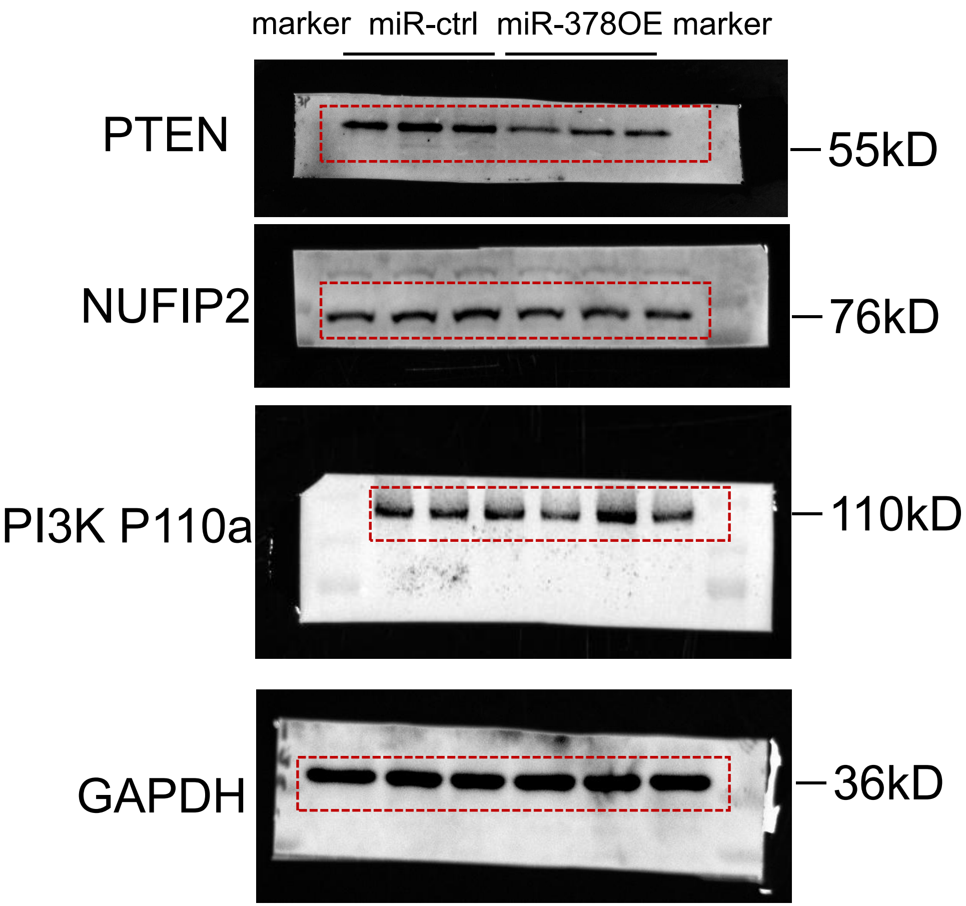

Full unedited gel for Figure 6J

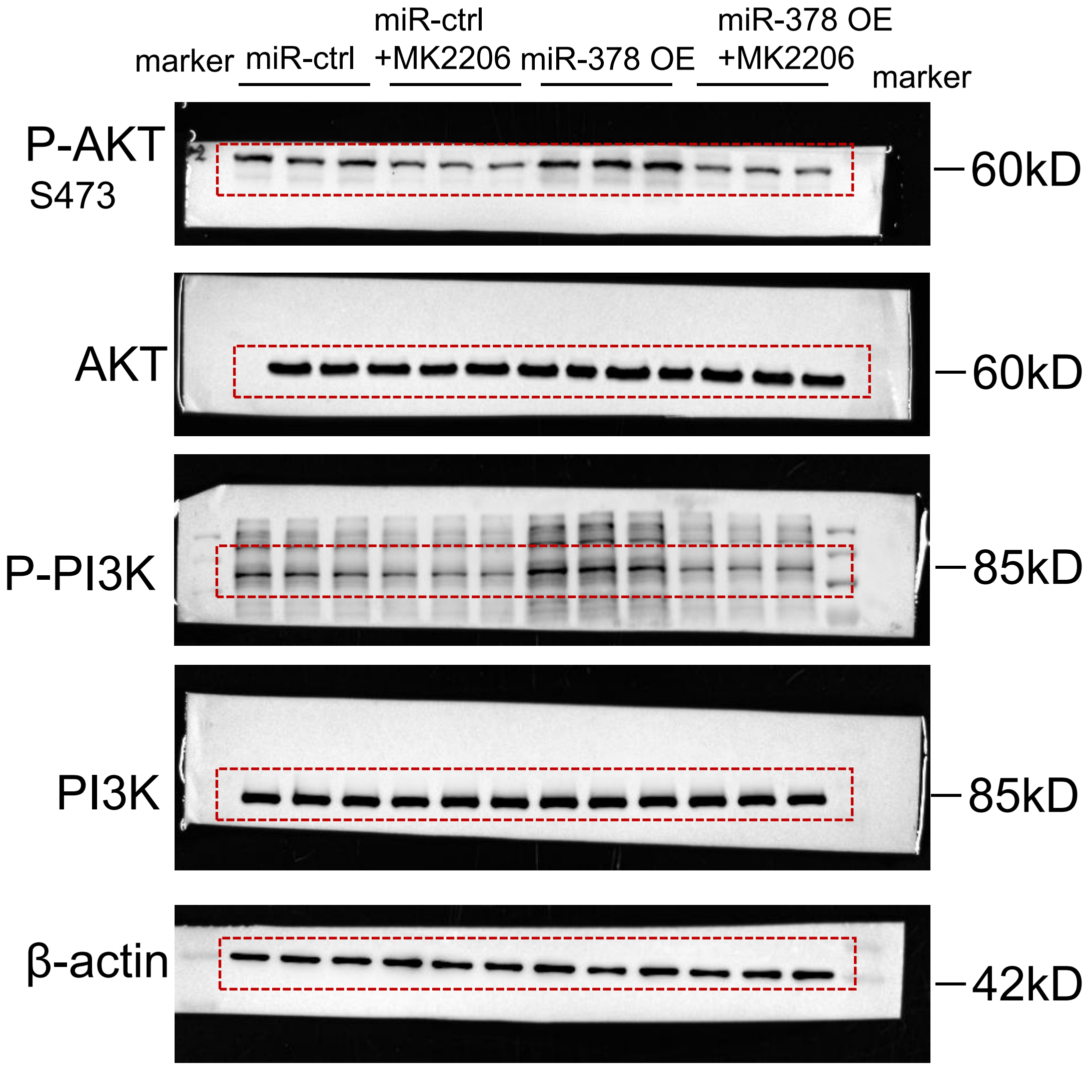

Full unedited gel for Figure S1F

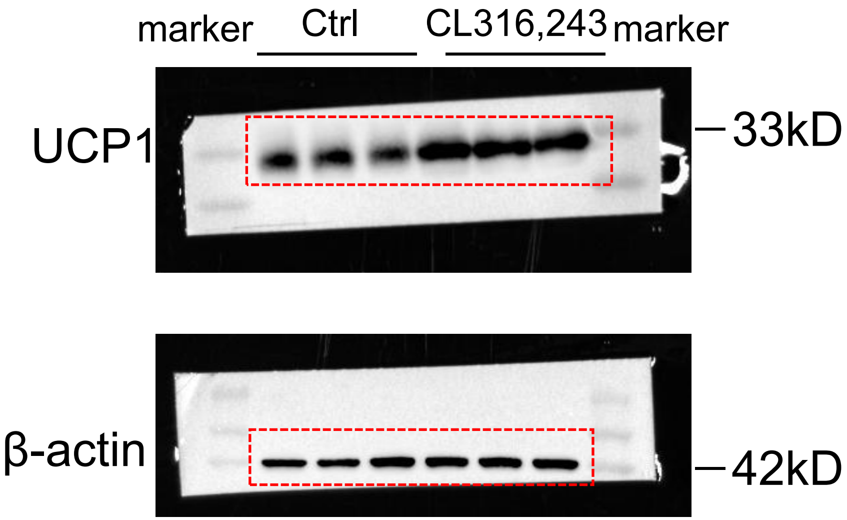

Full unedited gel for FigureS3A

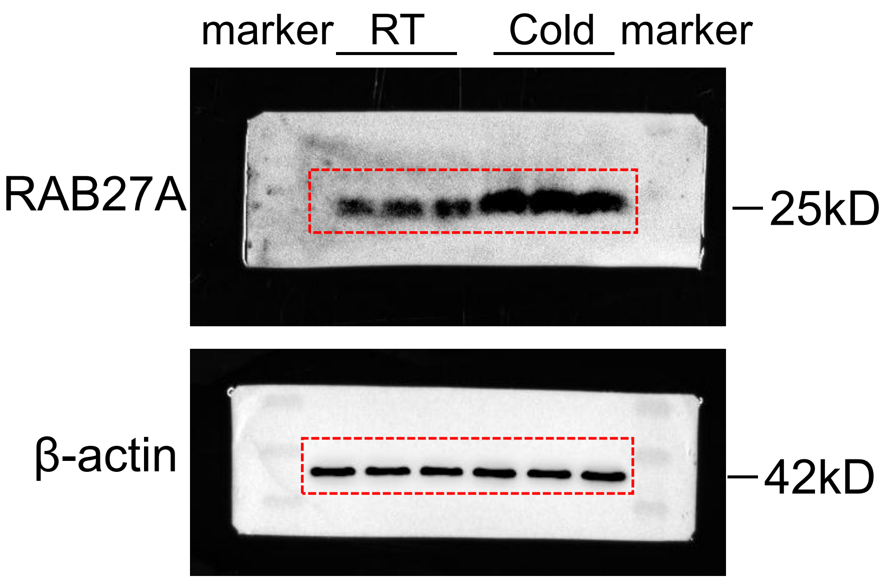

Full unedited gel for FigureS3B

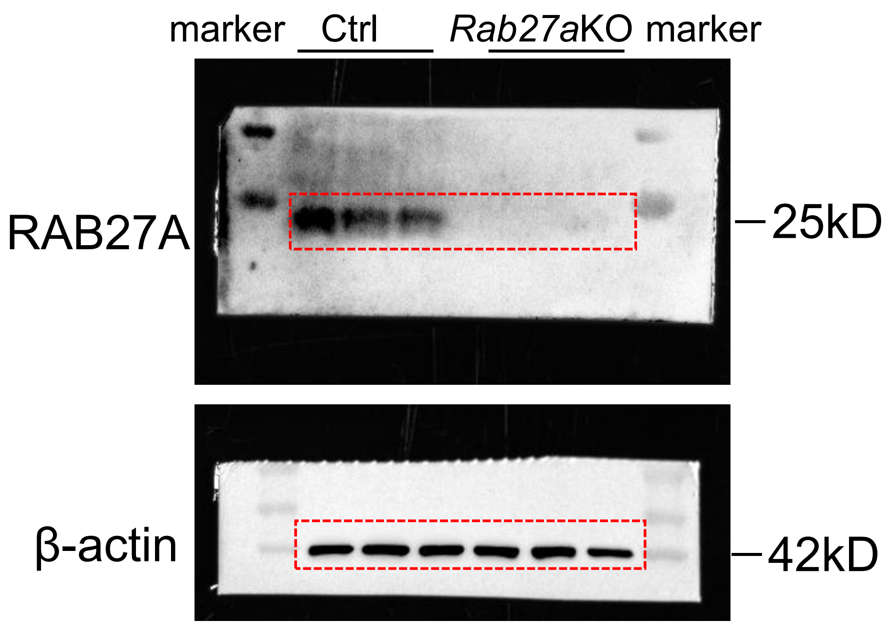

Full unedited gel for Figure S3C

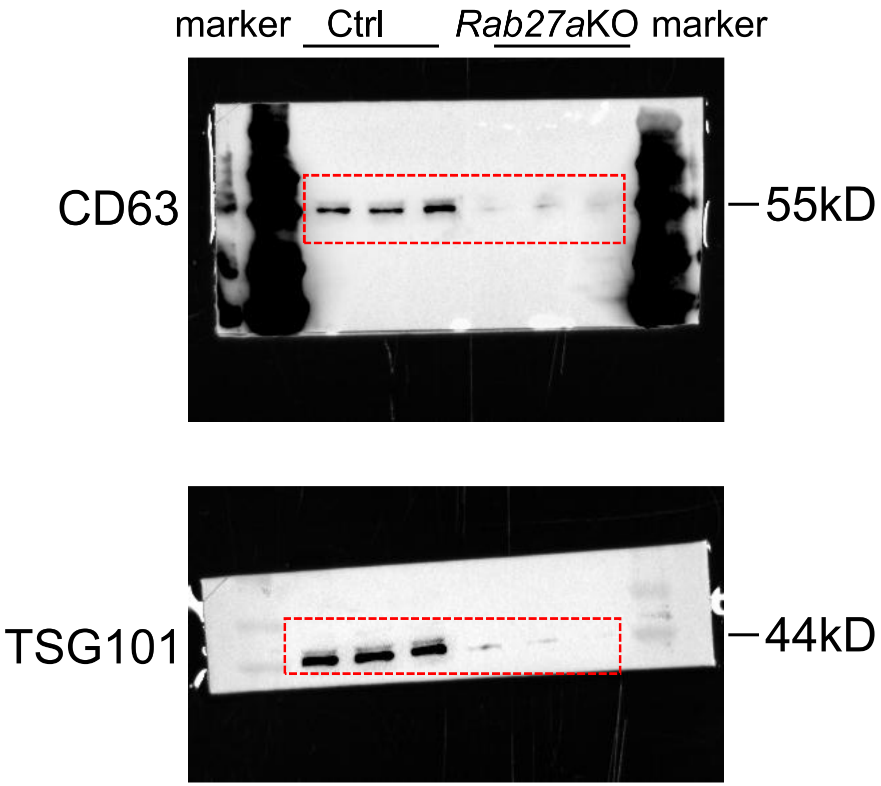

Full unedited gel for FigureS5D

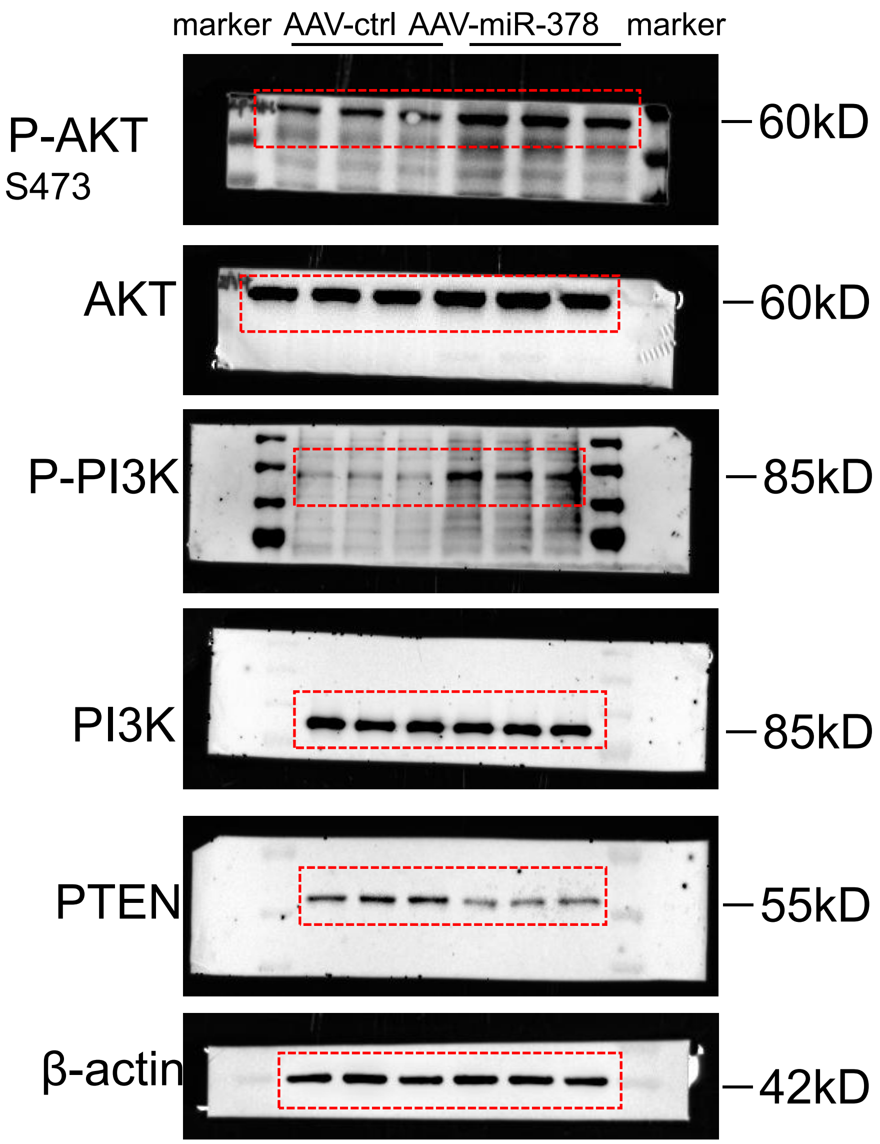

Full unedited gel for FigureS5H

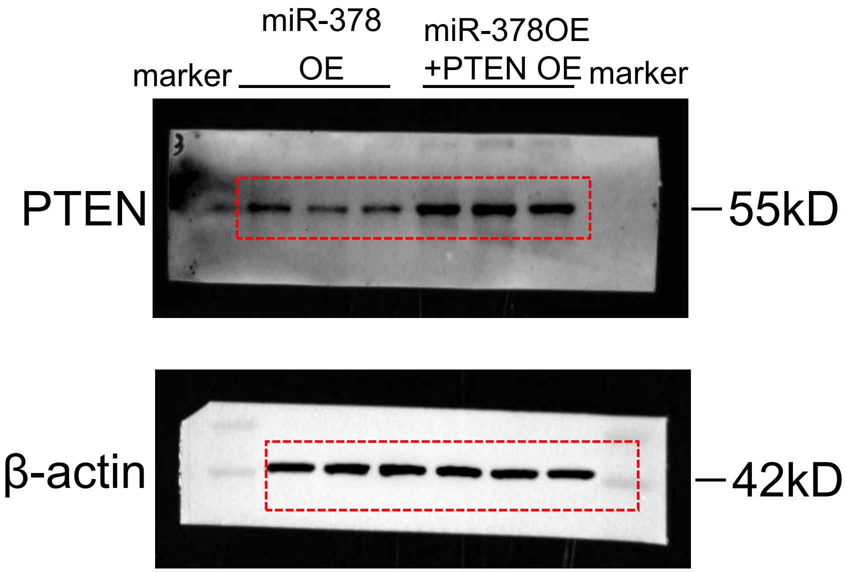

Supplement: Unedited blot and gel images [file jciinsight-11-201311-s298.pdf]
